# Transcriptional reprogramming of xylem cell wall biosynthesis in tension wood

**DOI:** 10.1093/plphys/kiab038

**Published:** 2021-02-02

**Authors:** Baoguang Liu, Juan Liu, Jing Yu, Zhifeng Wang, Yi Sun, Shuang Li, Ying-Chung Jimmy Lin, Vincent L Chiang, Wei Li, Jack P Wang

**Affiliations:** 1 State Key Laboratory of Tree Genetics and Breeding, Northeast Forestry University, Harbin 150040, China; 2 Department of Forestry, Beihua University, Jilin 132013, China; 3 Department of Life Sciences, College of Life Science, National Taiwan University, Taipei 10617, Taiwan; 4 Forest Biotechnology Group, Department of Forestry and Environmental Resources, North Carolina State University, Raleigh, North Carolina 27695

## Abstract

Tension wood (TW) is a specialized xylem tissue developed under mechanical/tension stress in angiosperm trees. TW development involves transregulation of secondary cell wall genes, which leads to altered wood properties for stress adaptation. We induced TW in the stems of black cottonwood (*Populus trichocarpa*, Nisqually-1) and identified two significantly repressed transcription factor (TF) genes: class B3 heat-shock TF (*HSFB3-1*) and *MYB092*. Transcriptomic analysis and chromatin immunoprecipitation (ChIP) were used to identify direct TF–DNA interactions in *P. trichocarpa* xylem protoplasts overexpressing the TFs. This analysis established a transcriptional regulatory network in which PtrHSFB3-1 and PtrMYB092 directly activate 8 and 11 monolignol genes, respectively. The TF–DNA interactions were verified for their specificity and transactivator roles in 35 independent CRISPR-based biallelic mutants and overexpression transgenic lines of *PtrHSFB3-1* and *PtrMYB092* in *P. trichocarpa*. The gene-edited trees (mimicking the repressed *PtrHSFB3-1* and *PtrMYB092* under tension stress) have stem wood composition resembling that of TW during normal growth and under tension stress (i.e., low lignin and high cellulose), whereas the overexpressors showed an opposite effect (high lignin and low cellulose). Individual overexpression of the TFs impeded lignin reduction under tension stress and restored high levels of lignin biosynthesis in the TW. This study offers biological insights to further uncover how metabolism, growth, and stress adaptation are coordinately regulated in trees.

## Teaser

PtrHSFB3-1 and PtrMYB092 mediate transcriptional reprogramming of secondary cell wall biosynthesis during tension wood formation by direct and indirect transcription factor–DNA interactions with regulatory specificity.

## Introduction

Wood is the most abundant terrestrial biomass on earth and essential raw material for timber and production of pulp and bio-based chemicals (Sarkanen, 1976). Wood formation in angiosperms occurs by differentiation of vascular cambium into fiber and vessel cells, accompanied by secondary wall thickening and programmed cell death (Esau, 1965). Under gravitational stimulus or mechanical stress induction, for example, by weights, cambium develops into a specialized tissue called tension wood (TW) on the upper side of branches and leaning stems (Côté et al., 1969; Scurfield, 1973). Compared with normal wood, TW has fewer vessel elements, increased cellulose quantity, and reduced lignin content (Timell, 1969; Sarkanen and Ludwig, 1971; Wu et al., 2000; Aoyama et al., 2001; Gerttula et al., 2015). Fiber cells are enriched in TW and the lumen of these cells contains a gelatinous layer (G-layer) of mostly crystalline cellulose (Côté et al., 1969). These physiological alterations affect the chemical and physical properties of wood and impact lignocellulosic conversion to chemicals and fiber products. TW improves pulp yield and enzymatic saccharification for biofuel production (Timell, 1986; Brereton et al., 2011; Sawada et al., 2018). Harnessing the plasticity of wood, particularly the unique properties of TW, brings revolutionary potential to advance the strategic improvement of wood for fiber and energy utilization. To implicate TW for lignocellulosic enhancement relies on gaining knowledge of its underlying molecular and genetic regulations.

TW formation involves complex networks of molecular and genetic interactions that transduce perceptions by phytohormone signaling to genetic reprogramming of xylogenesis (Wu et al., 2000; Andersson-Gunneras et al., 2006; Gerttula et al., 2015). Gravitational stimulation or mechanical stress triggers localized changes in auxin concentration associated with cambial differentiation for asymmetric radial growth in TW (Necesany, 1958; Leach and Wareing, 1967; Robnett and Morey, 1973). Immunolocalization of auxin polar transporter (*PIN3*) in TW of hybrid aspen (*P. alba* × *P. tremula*) indicates that endogenous auxin transports toward the cambial zone in TW and away from the cambium (toward the cortex) in the opposite wood (OW; Gerttula et al., 2015). Exogenous application of indole-3-acetic acid (IAA) induced TW formation in stems of hybrid poplar (Yu et al., 2017) and red maple (*Acer rubrum*) seedlings (Cronshaw and Morey, 1968), and the inhibition of auxin by *N*-1-naphthylphthalamic acid (NPA) suppressed TW formation (Yu et al., 2017). Gibberellic acid treatments promoted “gravibending” response and TW formation in hybrid aspen (Gerttula et al., 2015) and *Prunus spachiana* (Nakamura et al., 1994). Despite the importance of phytohormones, inherent challenges in modulating the spatiotemporal distribution of hormones in planta limit genomic and genetic approaches to revealing their molecular regulation in TW (Hellgren et al., 2004; Gerttula et al., 2015). Instead, much effort over the past decade has focused on the identification and characterization of differentially expressed genes (DEGs; Paux et al., 2005; Mutasa-Göttgens et al., 2012; Wang et al., 2014; Chen et al., 2015; Cai et al., 2018; Zinkgraf et al., 2018) and proteins (Bygdell et al., 2017) during TW formation.

TW formation by artificial stem-bending has been broadly applied to different angiosperm species, encompassing *P. tomentosa* (Wang et al., 2009; Chen et al., 2015), *P. tremula* (Bygdell et al., 2017), *Eucalyptus globulus* (Paux et al., 2005), *Liriodendron tulipifera* (Jin et al., 2011), *P. tremula* × *tremuloides* (Andersson-Gunneras et al., 2006), *P. alba* × *tremula* (Gerttula et al., 2015), and *Betula luminifera* (Cai et al., 2018). The induction of TW enabled genome-scale transcriptomic analyses to identify hundreds of DEGs specific to TW, including enzyme-encoding genes and transcription factors (TFs; Andersson-Gunneras et al., 2006; Wang et al., 2009; Jin et al., 2011; Chen et al., 2015; Gerttula et al., 2015). Typically, TW has reduced expression of genes involved in monolignol biosynthesis, polymerization (peroxidases and laccases), and xylan biosynthesis compared with normal wood and OW (Andersson-Gunneras et al., 2006; Chen et al., 2015). The expression of cellulose synthases and genes involved in uridine diphosphate glucose metabolism is elevated in TW (Paux et al., 2005; Andersson-Gunneras et al., 2006; Bygdell et al., 2017). Fasciclin-like arabinogalactan proteins, which mediate the regulation of cellulose microfibril angle and cell wall components adhesion, also increase in expression in TW (Plomion et al., 2001; Lafarguette et al., 2004; Kaku et al., 2009). These studies revealed the regulatory effects of tension stress on cell-wall component genes, but information on the direct or indirect transregulations that modulate these genetic changes remains lacking.

The transregulation of cell wall component genes in wood formation is mediated by TFs that function cooperatively and combinatorially in a transcriptional regulatory network (TRN; Lin et al., 2013, 2017; Chen et al., 2019; Yeh et al., 2019). TF expression profiles differ significantly between TW, normal wood, and OW (Chen et al., 2015). Artificial stem bending in *P. tomentosa* revealed 97 differentially expressed TFs specific to TW (Chen et al., 2015). Ethylene treatment-induced TW in *P. tremula* × *tremuloides* is associated with the differential expression of 168 TFs, including members in ethylene-induced ethylene response factor (ERF), basic helix–loop–helix (bHLH), v-myb avian myeloblastosis viral oncogene homolog (MYB), perforin domain-containing protein (MAC) WRKYGQK conserved sequence at N-terminal (WRKY), and ETHYLENE INSENSITIVE3-LIKE (EIN3-like) TF families (Vahala et al., 2013; Felten et al., 2018). Time-course analysis of TW formation in *P. alba* × *P. tremula* revealed clusters of temporally co-expressed TFs and cell wall genes (Zinkgraf et al., 2018). GA-induced TW formation in *P. alba* × *P. tremula* is sensitive to the transcript expression of a Class 1 KNOX homeodomain (ARK2) TF (Gerttula et al., 2015). Inference for TF-based reprogramming of cell wall biosynthesis in TW was made using genome-wide co-expression analyses (Ferreira et al., 2016). Six TFs (2 LIMs and 4 MYBs) were found to positively co-express with monolignol genes, and 10 TFs in the VNI, MYB, ASL, and KNAT families co-express with cellulose synthases during TW formation. However, the extent to which these TFs mediate the direct or indirect causal regulation of xylogenetic reprogramming in TW remains to be elucidated.

Relationships between TFs and their target genes are best evaluated by quantitative determination of the regulatory specificity and direct TF–DNA interactions in a hierarchical TRN. We recently established a four-layer TRN associated with wood formation in *P. trichocarpa* that describes how a key TF (PtrSND1-B1; secondary wall-associated NAC-domain protein) transregulates 17 xylem-specific TFs and 27 cell wall component biosynthetic genes (Chen et al., 2019). The TRN for wood formation was made possible due to advances in the development of woody tissue-specific chromatin-immunoprecipitation (ChIP) method and stem xylem protoplast system (Lin et al., 2013, 2014; Li et al., 2014). The wood formation TRN is represented by 57 direct TF–DNA interactions and 9 TF protein complexes, determined by a top-down integrative approach using TF-epitope ChIP transregulation, yeast two-hybrid (Y2H), and bimolecular fluorescence complementation (BiFC; Chen et al., 2019).

In this study, we focused on the transcriptional reprogramming of lignin biosynthesis and the effects on wood formation under tension stress. We show that two TFs, *PtrHSFBb3-1* (heat shock TF, HSF) and *PtrMYB092* (v-myb avian myeloblastosis viral oncogene homolog), reprogram the wood formation TRN by suppressing monolignol biosynthesis during TW formation. *PtrHSFBb3-1* and *PtrMYB092* were the two most significant TW responsive TFs based on transcriptomic analysis and artificial stem bending in *P. trichocarpa*. We used ChIP coupled with qPCR and full transcriptome RNA-seq to reveal the direct TF–DNA transregulatory effects of *PtrHSFBb3-1* and *PtrMYB092* on monolignol biosynthetic genes and established a TW-specific TRN. This TRN and its transregulatory specificity and effects were then validated by CRISPR-based genome editing and overexpression of *PtrHSFBb3-1* and *PtrMYB092* in *P. trichocarpa*. Artificially induced tension stress was used to further support the TF-functions in TW of transgenics and wild-type. We discovered a TW-specific TRN mediated by *PtrHSFB3-1* and *PtrMYB092* that transactivates the expression of 31 genes involved in the biosynthesis of lignin, cellulose, and hemicelluloses, and that this TW-TRN is independent of the TRN for normal wood formation (Chen et al., 2019).

## Results

### Tension stress alters cell-wall biosynthesis and transcriptome in stem differentiating xylem of *P*. *trichocarpa*

We induced the formation of TW in the stem of 6-month-old greenhouse-grown *P*. *trichocarpa* by bending the stem at a 90-degree angle (Supplemental Figure S1). After bending for 21 d, the stem differentiating xylem (SDX) tissue of the upper bent region, the TW, was analyzed for changes in chemical composition. The TW showed a substantial increase in cellulose (represented by ∼31% increase in glucose) and a drastic reduction in lignin content by ∼39% compared with normal stem wood (Supplemental Table S1). These alterations in wood properties are characteristic of TW in field-grown trees, suggesting the simple bending treatment is a useful system to generate and study TW formation (Wu et al., 2000; Lu et al., 2005). We then analyzed the transcriptome of *P*. *trichocarpa* in response to time-course TW formation. A 90-degree bending was made to 6-month-old *P*. *trichocarpa* plants (Supplemental Figure S1) for 3, 7, 14, and 21 d, and the bent stems were harvested for morphological analysis (Supplemental Figures S2, S3) and RNA collection. G-layer in fiber cells started to emerge after 3-d bending and became more visible after 7 d (Supplemental Figures S2, S3), suggesting the initiation of TW formation at these time points, where a strong transcriptional response to TW formation would be expected. We then used the RNAs from the control and the 3- and 7-d bending experiments for transcriptomic analysis. TW formation for 3 and 7 d induced 391 and 2,470 DEGs (fold change > 2 or < 0.8, FDR < 0.05), respectively, compared with the 0-day (no bending) control (Supplemental Datasets S1, S2).

### TW-specific expression of genes for cell-wall component biosynthesis and TF genes

**Figure 1:**
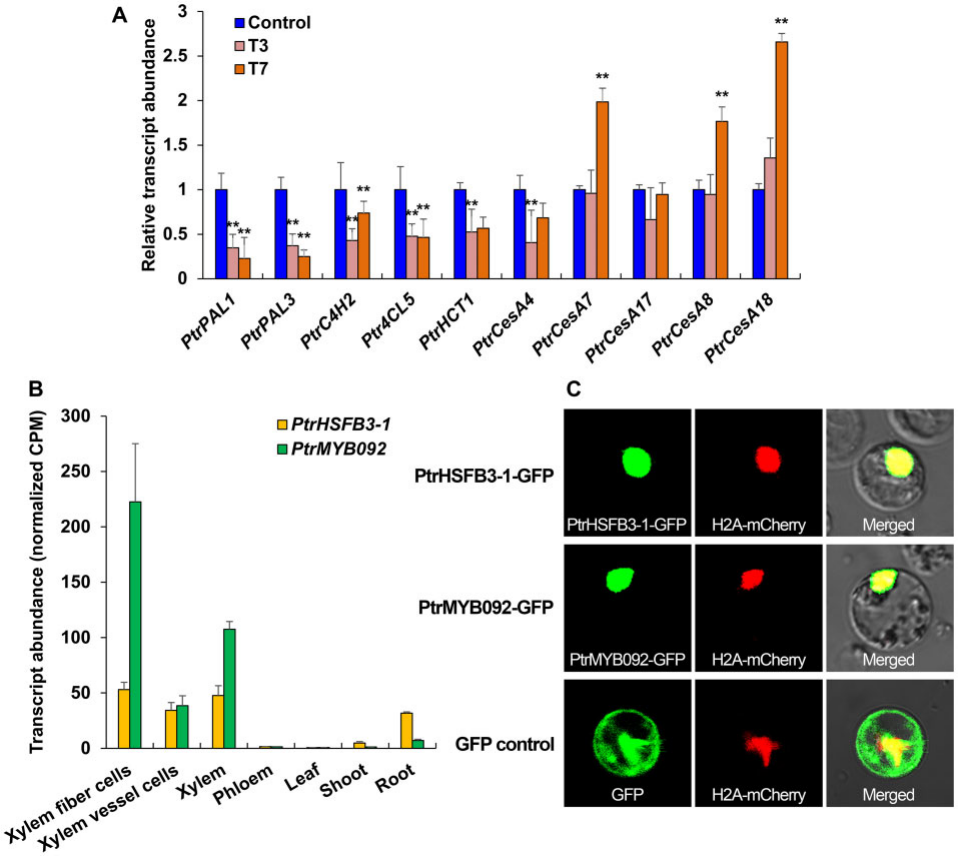
*PtrHSFB3-1* and *PtrMYB092* transcript expression profiles, subcellular localizations, and tension-stress responsive expression of cell wall component biosynthetic genes. A, Relative transcript abundance of cell wall component biosynthetic genes (*x*-axis) in response to 3- and 7-d stem bending (T3 and T7, respectively) in SDX of *P. trichocarpa*. Control represents vertical (no bending) stem. Error bars depict one standard error (SE) of three biological replicates. **P-values < 0.01 (Student’s *t* test). B, Transcript expression profiles (normalized CPM) of *PtrHSFB3-1* and *PtrMYB092* in five different tissues (xylem, phloem, leaf, shoot, and root) and two xylem cell types (fiber and vessel cells) in *P. trichocarpa*. Error bars represent one SE of three biological replicates. C, Subcellular protein localization of PtrHSFB3-1 and PtrMYB092. C-terminus GFP fusions of PtrHSFB3-1 and PtrMYB092 were individually co-transferred with a nuclear marker (H2A-mCherry) into *P. trichocarpa* SDX protoplasts for confocal microscopy. The co-transfer of GFP and H2A-mCherry into SDX protoplasts was included as the control. From left to right panels, GFP signal, mCherry signal, and merged signals of GFP and mCherry.

To understand how TW formation alters lignin and cellulose biosynthesis, we focused on screening TW formation DEGs for genes involved in lignin and cellulose biosynthesis and TF genes that may transregulate such cell-wall genes for the alteration. We found that the expression of five monolignol biosynthetic pathway genes encoding *PtrPAL1*, *PtrPAL3*, *PtrC4H2*, *Ptr4CL5*, and *PtrHCT1* enzymes were repressed after 3- and 7-d TW formation (Figure 1, A). Our previous work demonstrated that downregulating the transcript abundance of these monolignol genes would result in lignin reduction in *P*. *trichocarpa* (Wang et al., 2018, 2019). Therefore, the repression of these five genes is consistent with the characteristics of low lignin in TW. Of the five secondary cell-wall cellulose synthase genes, three (*PtrCesA7*, *PtrCesA8*, and *PtrCesA18*) were activated after 7-d bending (Figure 1, A), consistent with the high cellulose content in TW.

We then identified TF genes from the DEGs that may mediate low lignin and high cellulose formation in TW. We observed 167 putative TF DEGs (fold change > 2 or < 0.8, FDR < 0.05) in 3- and 7-d TW compared with the control (Supplemental Datasets S1, S2). Of these DEGs, 61 were differentially repressed and represent candidate TFs for regulating TW formation, because the repression of these TFs would likely cause the repressed expression of monolignol genes. The repressed TFs may also be direct repressor or indirect activator mediating cellulose biosynthesis. From the 61 repressed TF DEGs, we selected two of the most significantly differentially repressed ones for further study. One is a member of the *P. trichocarpa* Class B3 HSF (*PtrHSFB3-1*, Potri.006G049200), of which expression was reduced by 3.0-fold (FDR ≤ 0.01) after 3-d bending (T3) and by 2.9-fold (FDR ≤ 0.01) at T7 (Supplemental Datasets S1, S2). The other is *PtrMYB092* (Potri.001G118800, 2.7-fold reduction in T3, FDR ≤ 0.01; 3.3-fold reduction in T7, FDR ≤ 0.01; Supplemental Datasets S1, S2 and Supplemental Figure S4).

*PtrHSFB3-1* and *PtrMYB092* are preferentially and abundantly expressed in stem xylem relative to other tissues examined, and the abundance is attributed to the expression in both fiber and vessel cells, the two major wood formation cells (Figure 1, B; Shi et al., 2017). *PtrHSFB3-1* is one of four HSF genes expressed more specifically in SDX of *P*. *trichocarpa* (Shi et al., 2017). Homologs of *PtrHSFB3-1* in other plant species were shown to respond to biotic and abiotic stress (Scharf et al., 2012; Neu et al., 2019), but their involvement in the regulation of xylem development has not been previously reported.

In a previous study, *PtoMYB92*, a homolog of *PtrMYB092* in *P. tomentosa*, was overexpressed in transgenic plants and shown to activate the expression of monolignol biosynthetic genes, *PAL4*, *C4H2*, *C3H3*, *4CL5*, *HCT1*, *CCOAOMT1*, *CCR2*, *AldOMT2*, and *CAD1* (Li et al., 2015). It is unknown whether PtoMYB92 can directly activate these genes or not. PtoMYB92 could transactivate three (*C3H3*, *CCOAOMT1*, and *CCR2*) of these nine monolignol genes in Arabidopsis leaves (Li et al., 2015).

### Subcellular localization reveals that *PtrHSFB3-1* and *PtrMYB092* are nuclear proteins

For *PtrHSFB3-1* and *PtrMYB092* to function as TFs associated with TW formation, the TFs need to be expressed in time and place that is consistent with the transregulation of cell wall biosynthetic genes. We have shown that *PtrHSFB3-1* and *PtrMYB092* gene expressions are temporally (TW-specific, Supplemental Datasets S1, S2) and spatially (SDX specific, Figure 1, B) consistent with regulation of TW formation. We next determined the subcellular localization of *PtrHSFB3-1* and *PtrMYB092* in vivo. Transient expression vectors (*pUC19-35S-PtrHSFB3-1-sGFP* and *pUC19-35S-PtrMYB092-sGFP*) were assembled to express each TF as a protein–GFP fusion. The vectors were individually co-transfected with H2A*-*mCherry (a nuclear-localization marker) into SDX protoplasts of *P. trichocarpa*. After 7-h incubation, green fluorescence signals of GFP-fused *PtrHSFB3-1* and *PtrMYB092* were observed to co-localize with the red fluorescence signals of H2A-mCherry, indicating nucleus localization of the TFs (Figure 1, C). The fluorescence pattern of the GFP only control was uniformly distributed (Figure 1, C) in the protoplasts and distinct from the fluorescence pattern of H2A-mCherry. The nucleus localization of *PtrHSFB3-1* and *PtrMYB092* in SDX cells is consistent with their functions as TFs in these nuclei. We next determined if these two TFs can transregulate the expression of genes involved in secondary cell wall component biosynthesis for TW formation.

### *PtrHSFB3-1* and *PtrMYB092* transactivate monolignol biosynthetic pathway genes in stem xylem protoplasts

To determine if *PtrHSFB3-1* and *PtrMYB092* can transregulate the expression of cell wall component genes, we used a transient overexpression system in SDX protoplasts of *P. trichocarpa* coupled with RT-qPCR (Lin et al., 2013, 2014; Chen et al., 2019; Li et al., 2019). *PtrHSFB3-1* and *PtrMYB092* coding sequences were cloned into *pUC19-35S-RfA-35S-sGFP* to produce transient overexpression vectors (*pUC19-35S-PtrHSFB3-1-35S-sGFP* and *pUC19-35S-PtrMYB092-35S-sGFP*). These overexpression vectors and a control vector that does not contain a TF (*pUC19-35S-sGFP*) were individually transfected into SDX protoplasts. We then quantified the levels of *PtrHSFB3-1* and *PtrMYB092* overexpression and, as a preliminary test, the overexpression effects on the transcript abundances of six randomly selected monolignol genes (*PtrCAld5H2*, *PtrAldOMT2*, *PtrHCT1*, *PtrCCoAOMT1*, *PtrCCoAOMT2*, and *PtrCCoAOMT3*). *PtrHSFB3-1* and *PtrMYB092* transcripts were overexpressed by 1,503 and 442 folds, respectively, in the transfected SDX protoplasts (Supplemental Figure S5, A). *PtrHSFB3-1* overexpression significantly transactivated (fold change > 2; *P *<* *0.01) the expression of *PtrCCoAOMT2* (Supplemental Figure S5, B). The overexpression of *PtrMYB092* significantly transactivated (fold change > 2; *P *<* *0.01) the expression of four monolignol genes (*PtrAldOMT2*, *PtrHCT1*, *PtrCCoAOMT1*, and *PtrCCoAOMT2*; Supplemental Figure S5, B). Having shown that *PtrHSFB3-1* and *PtrMYB092* are involved in regulating cell-wall biosynthesis, we next discovered the genome-wide transregulatory functions of *PtrHSFB3-1* and *PtrMYB092* using full transcriptome analysis of *P. trichocarpa* SDX protoplasts overexpressing with these two TFs.

### Genome-wide analysis of *PtrHSFB3-1* and *PtrMYB092* transregulation in stem xylem cells

*PtrHSFB3-1* (*pUC19-35S-PtrHSFB3-1-35S-sGFP*), *PtrMYB092* (*pUC19-35S-PtrMYB092-35S-sGFP*), and *pUC19-35S-sGFP* (control) were individually overexpressed in *P. trichocarpa* SDX protoplasts and the transfected protoplasts were sequenced (Supplemental Figure S6) by full transcriptomics to identify DEGs. *PtrHSFB3-1* overexpression significantly altered the transcript abundances of 1,322 genes compared with the control (Supplemental Dataset S3), with 1,072 upregulated and 250 downregulated DEGs (fold change > 2 or < 0.8; FDR < 0.05). Overexpression of *PtrMYB092* significantly altered the transcript abundances of 1,054 genes (Supplemental Dataset S4), with 985 upregulated and 69 downregulated DEGs (fold change > 2 or < 0.8, FDR < 0.05). The high ratio of up-regulated DEGs to down-regulated DEGs suggests that *PtrHSFB3-1* and *PtrMYB092* are likely to be transcriptional activators, consistent with our preliminary transregulatory assays (Supplemental Figure S5, B).

**Figure 2:**
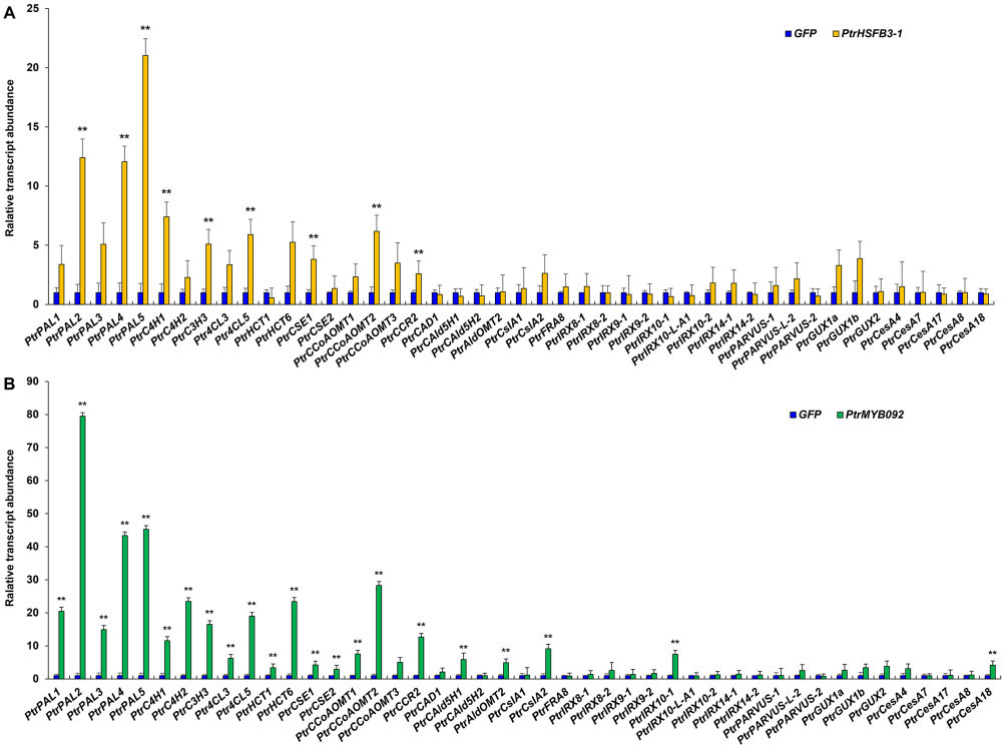
Changes in transcript expression of cell wall component biosynthetic genes in response to overexpression of PtrHSFB3-1 and PtrMYB092 in SDX protoplasts. Relative transcript abundance of cell wall component biosynthetic genes in *P. trichocarpa* SDX protoplasts overexpressing PtrHSFB3-1 (A) and PtrMYB092 (B), compared with control SDX protoplasts overexpressing GFP. The average expression level of three biological replicates is presented. Error bars depict one standard error of three biological replicates. **P-values < 0.01, Student’s *t* test.

Of the 1,322 DEGs of *PtrHSFB3-1*, 9 are associated with cell wall biosynthesis. These nine genes are all monolignol biosynthetic genes, and all were activated (Figure 2, A, **P <* *0.01, genes ID showed in Supplemental Dataset S5). Of the 1,072 DEGs of *PtrMYB092*, 22 are associated with cell-wall biosynthesis, which include 19 monolignol (**P <* *0.01, Figure 2, B, genes ID in Supplemental Dataset S5), 1 cellulose (*PtrCesA18*, Potri.004G059600, Figure 2, B), and 2 hemicelluloses (*PtrCslA2*, Potri.010G234100 and *PtrIRX10-1*, Potri.001G068100, **P <* *0.01, Figure 2, B) biosynthetic genes. All these 22 genes were activated. Gene ontology (GO) analysis (G:profiler, http://biit.cs.ut.ee/gprofiler/; Reimand et al., 2016) of the remaining DEGs revealed an association with many biological processes related to wood formation. For example, *PtrHSFB3-1* overexpression transactivated genes belonging to the phenylpropanoid biosynthetic process and secondary metabolite biosynthetic process (Supplemental Dataset S6). *PtrMYB092* overexpression transactivated genes belonging to the l-phenylalanine metabolic process and cinnamic acid biosynthetic process (Supplemental Dataset S7). To determine if the transactivation of *PtrHSFB3-1* and *PtrMYB092* on cell wall component genes are based on direct TF–target DNA interactions or indirect regulatory effects, we next performed chromatin immunoprecipitation (ChIP) of the TF–DNA interactions using SDX protoplasts.

### *PtrHSFB3-1-sGFP* and *PtrMYB092-sGFP* fusion proteins retain their native transregulatory activities

ChIP is one of the most robust methods to identify individual or genome-wide direct TF–target DNA interactions in vivo (Solomon et al., 1988; Kim and Ren, 2006; Farnham, 2009). We have recently developed a ChIP system specific to the wood-forming tissue of *P. trichocarpa* and other forest tree species (Lin et al., 2013; Li et al., 2014, 2019; Chen et al., 2019) and used it to reveal complex hierarchical transcriptional networks of direct TF–target DNA interactions that regulate secondary cell wall biosynthesis (Lin et al., 2013; Chen et al., 2019). In this system, the TF as a GFP-fusion is transfected into *P. trichocarpa* SDX protoplasts and overexpressed. Following cross-linking of the TF–GFP to their bound promoter regions, an anti-GFP antibody was used to purify the TF–DNA complexes to identify and quantify (ChIP qPCR or ChIP-seq) the specific TF-enriched promoters of the target genes (Mukhopadhyay et al., 2008; Chen et al., 2019; Li et al., 2019).

To ensure that the fusion of GFP does not interfere with the native transregulatory functions of *PtrHSFB3-1* and *PtrMYB092*, we assayed the transactivation activity of the TFs with and without GFP fusion. Overexpression vectors for the TF–GFP fusions (*pUC19-35S-PtrHSFB3-1-sGFP* and *pUC19-35S-PtrMYB092-sGFP*) and without the GFP fusion (*pUC19-35S-PtrMYB092-35S-sGFP* and *pUC19-35S-PtrHSFB3-1-35S-sGFP*) were individually transfected into SDX protoplasts. The levels of transactivation were then measured by RT-qPCR for five monolignol genes (*PtrHCT1*, *PtrAldOMT2*, *PtrCCoAOMT1*, *PtrCCoAOMT2*, and *PtrCCoAOMT3*) randomly selected from those being activated by *PtrHSFB3-1* and *PtrMYB092* (Supplemental Figure S7). An empty vector without TF (*pUC19-35S-sGFP*) was included as the control. For both *PtrHSFB3-1* and *PtrMYB092*, the levels of transactivation of target genes were very similar between the TFs with and without GFP fusion (Supplemental Figure S7), suggesting that the TF–GFP fusions retain the TF’s native regulatory functions. Previously, we had also shown that GFP fusion does not affect TF’s native transactivation functions (Chen et al., 2019).

### PtrHSFB3-1 and PtrMYB092 mediate direct TF–DNA transregulation of cell wall component biosynthetic genes

**Figure 3:**
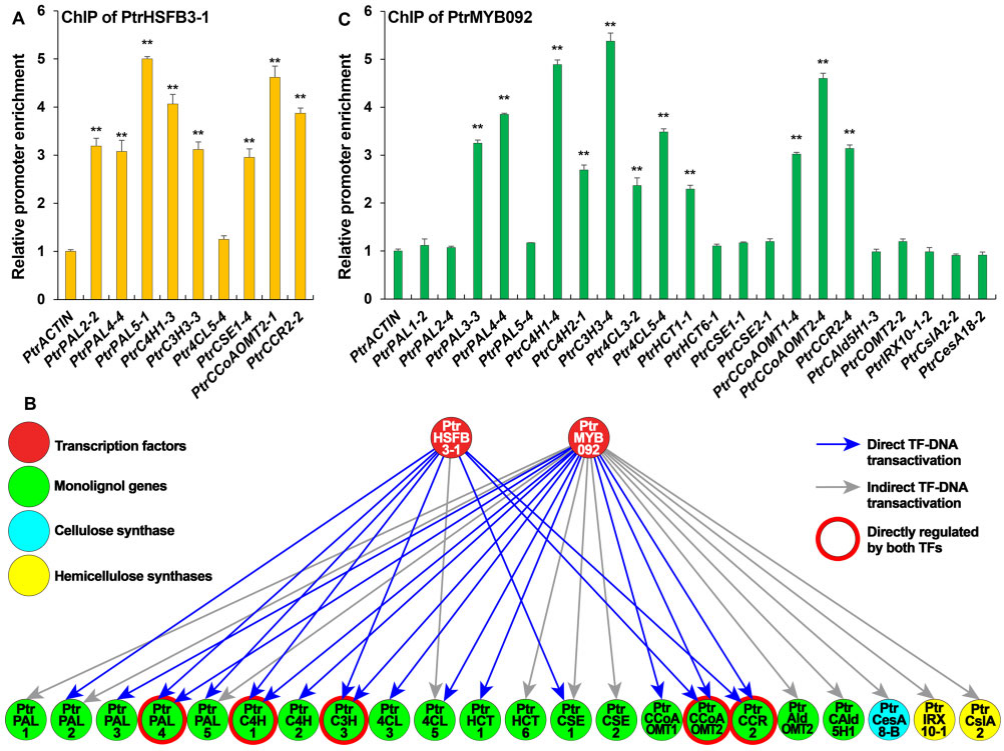
PtrHSFB3-1 and PtrMYB092 mediated TRN for cell wall component biosynthesis. ChIP reverse transcription quantitative PCR (RT-qPCR) identification of cell wall biosynthetic genes directly targeted by PtrHSFB3-1 (A) and PtrMYB092 (B) PtrHSFB3-1 and PtrMYB092 mediated a TW-TRN for transregulation of cell-wall component biosynthetic genes in *P. trichocarpa*. The two TFs and 22 cell wall biosynthetic genes (gene IDs shown in Supplemental Dataset S7) are connected by 19 direct (blue lines) and 12 indirect (gray lines) TF–DNA interactions. PtrHSFB3-1 and PtrMYB092 have five common direct target genes (red rings). Arrows indicate positive regulations (transactivation). C, The number following each gene name represents the relative location of the promoter regions (Supplemental Dataset S8) with the highest efficiency of ChIP enrichment. TF–DNA interaction is considered positive if promoter enrichment > two-fold. The promoter of *PtrACTIN* was assayed as the control and set to 1. Three independent biological replicates for each ChIP assay were performed and the average values of the replicates are presented in the bar charts. Error bars represent one the standard error (SE) of three biological replicates. **P-values < 0.01 (Student’s *t* test).

We then used this system to perform ChIP on the TF–GFP-transformed protoplasts using anti-GFP antibodies to identify cell wall component genes directly transregulated by *PtrHSFB3-1* and *PtrMYB092*. Overexpression vectors to express the TFs as C-terminal GFP-fusions (*pUC19-35S-PtrHSFB3-1-sGFP* and *pUC19-35S-PtrMYB092-sGFP*; Chen et al., 2019) were constructed and individually transfected into *P. trichocarpa* SDX protoplasts. Following cross-linking and anti-GFP antibody purification, qPCR was performed for four fragments of the ∼2,000 bp chromatin DNA fragments (promoter sequences) upstream of the coding region of each tested cell-wall component genes, with qPCR products ranging from 100 to 200 bp. Of the nine monolignol biosynthetic genes that were activated by PtrHSFB3-1 (Figure 2, A, **P <* *0.01), eight were confirmed by ChIP qPCR to be PtrHSFB3-1’s direct targets (Figure 3, A, **P <* *0.01 and Figure 3, B). For *PtrMYB092*, 11 of the 22 activated cell-wall component genes were confirmed by ChIP qPCR to be direct targets (Figure 3, C, **P <* *0.01, and Figure 3, B). All these 11 direct targets of PtrMYB092 are also monolignol genes. Three significantly activated polysaccharide biosynthesis genes (*PtrCesA18*, *PtrIRX10-1*, and *PtrCslA2*, Figure 2, B) are indirect targets (Figure 3, C). In summary, PtrHSFB3-1 and PtrMYB092 directly target a total of 14 unique monolignol genes, which include five common targets, *PtrPAL4*, *PtrC4H1*, *PtrC3H3*, *PtrCCoAOMT2*, and *PtrCCR2* (Figure 3, A and C, **P <* *0.01, and Figure 3, B). The unique and common targets indicate that *PtrHSFB3-1* and *PtrMYB092* have both independent and cooperative regulatory functions in mediating the transcriptional reprogramming for TW formation. We next altered the expression of *PtrHSFB3-1* and *PtrMYB092* in transgenic *P*. *trichocarpa* to test their regulatory roles in vivo.

### CRISPR-based editing of *PtrHSFB3-1* and *PtrMYB092* reduces lignin content, increases cellulose quantity, and improves plant growth in *P. trichocarpa*

Our analysis of TW formation implied that tension stress-induced repression of *PtrHSFB3-1* and *PtrMYB092* (Supplemental Datasets S1, S2) caused reduced lignification and augmented cellulose biosynthesis (Supplemental Table S1). To verify these transregulatory effects, we generated loss-of-function mutants of the two TFs in *P. trichocarpa* using CRISPR-based editing with *Streptococcus pyogenes* Cas9 (Deltcheva et al., 2011; Jiang et al., 2013; Heler et al., 2015). sgRNAs were designed to target SNP-free regions in the exons of *PtrHSFB3-1* and *PtrMYB092* (see the “Materials and methods” section; Supplemental Figure S8). The designed sgRNAs were validated for in vitro cleavage of target DNA using recombinant SpCas9-sgRNA ribonucleoprotein (RNP) cleavage assays (Supplemental Figure S9). The RNPs were able to cleave 100% of their target DNA to the correct sized fragments (Supplemental Figure S9), confirming the specificity and functions of the sgRNAs for editing *PtrHSFB3-1* and *PtrMYB092*. The validated sgRNAs were then individually cloned into the *pEgP237-2A-GFP* vector (Osakabe et al., 2016; Ueta et al., 2017) for CRISPR-based editing of the TFs using our improved *P. trichocarpa* transformation protocol (Song et al., 2006; Li et al., 2019). From the CRISPR-edited transgenic lines, we selected three independent biallelic mutants for *PtrHSFB3-1* and three for *PtrMYB092*. All six biallelic mutant lines induced the complete loss-of-function of the target TFs. The three mutant lines consisted of two heterozygous (*KO-PtrHSFB3-1-5-1* and *KO-PtrHSFB3-1-5-2*) and one homozygous (*KO-PtrHSFB3-1-5-3*) edits for *PtrHSFB3-1*, and one heterozygous (*KO-PtrMYB092-15-2*) and two homozygous (*KO-PtrMYB092-15-1* and *KO-PtrMYB092-15-3*) edits for *PtrMYB092* (Supplemental Figure S10).

**Figure 4:**
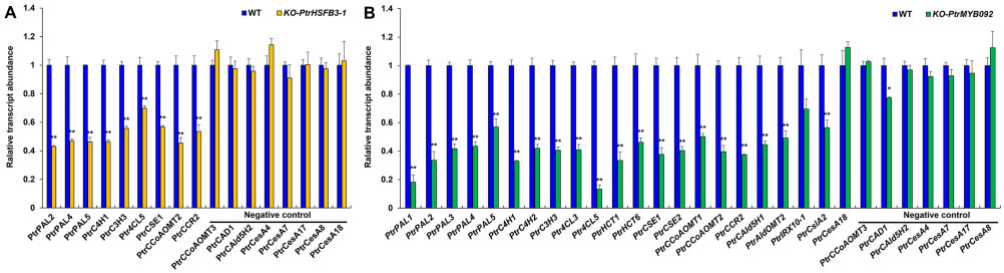
Loss-of-function genome editing of *PtrHSFB3-1* and *PtrMYB092* in *P. trichocarpa* on transcript expression of target cell wall component biosynthetic genes. CRISPR-based editing of (A) *PtrHSFB3-1* (*KO-PtrHSFB3-1*) and (B) *PtrMYB092* (*KO-PtrMYB092*) for loss-of-function in transgenic *P. trichocarpa* and RT-qPCR quantification of their direct and indirect target gene expressions in SDX. Blue bars represent the relative transcript abundances of the target genes in the wild-type *P. trichocarpa*, set to 1. Negative controls denote cell wall component biosynthetic genes not transregulated by PtrHSFB3-1 (A) or PtrMYB092 (B). Error bars represent one SE of three biological replicates (independent transgenic lines). *P-values < 0.05, **P-values < 0.01 (Student’s *t* test).

The transcript abundance of all cell-wall component genes regulated by PtrHSFB3-1 (eight direct targets and one indirect target, Figure 3, B) was significantly reduced (*P *<* *0.01) in SDX of the mutants compared with wild-type (Figure 4, A). Cell wall genes not regulated by PtrHSFB3-1 (represented by a few monolignol genes and all secondary cell-wall *CesA* genes) did not change in transcript abundance in the *PtrHSFB3-1* mutants (negative controls in Figure 4, A). Similarly, for *PtrMYB092* mutant lines, the transcript abundances of all 11 direct targets and 9 of the 11 indirect targets (Figure 3, B) were significantly reduced (*P *<* *0.01) compared with the wild-type (Figure 4, B). The expression of *PtrMYB092*’s remaining two indirect target genes (*PtrIRX10-1* and *PtrCesA18*) was not significantly reduced in the *PtrMYB092* mutants (Figure 4, B), suggesting the involvement of other unknown regulatory mechanisms. The reduced expression of all direct target genes of *PtrHSFB3-1* and *PtrMYB092* in the mutant lines (Figure 4) is consistent with the protoplast-derived functions (Figure 2) of these TFs as transactivators for cell-wall component biosynthesis.

**Figure 5:**
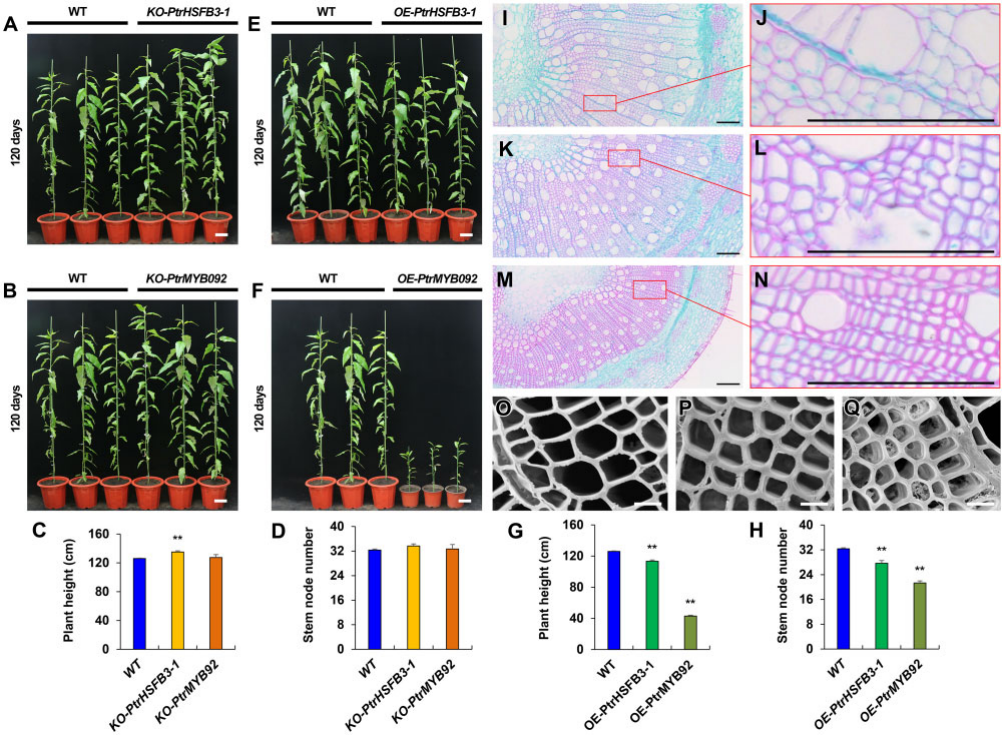
Knockout (KO) and overexpression (OE) of *PtrHSFB3-1* and *PtrMYB092* genes in transgenic *P. trichocarpa* and the impacts thereof on tree growth, number of stem internodes, and secondary wall thickness in xylem cells. Relative height (white bars = 10 cm) of 120-d-old greenhouse grown transgenic (A) *KO-PtrHSFB3-1*, (B) *KO-PtrMYB092*, (E) *OE- PtrHSFB3-1*, and (F) *OE-PtrMYB092* compared with wild-type (WT) *P. trichocarpa*. Line IDs are ordered from left to right as WT1, 2, and 3, (A) *KO-PtrHSFB3-1-5-1*, *-2*, and *-3*, (B) *KO-PtrMYB092-15-1*, *-2*, and *-3*, (E) *OE-PtrHSFB3-1-4*, *-7*, and *-11*, and (F) *OE-PtrMYB092-6*, *-7*, and *-9*. Statistical analysis of plant height (C, G) and stem internode numbers (D, H). Error bars represent one SE of three biological replicates (independent transgenic lines). **P < 0.01 (Student’s *t* test). Stem cross-sections (9th internode) of wild type (I, J), and transgenic *OE-PtrHSFB3-1* (K, L) and *OE-PtrMYB092* (M, N) (black bars = 100 μm). (O–Q) Scanning electron micrographs (SEM) of wild type (O), *OE-PtrHSFB3-1* (P), and *OE-PtrMYB092*. (Q) Stem cross-sections (9th internode; white bars = 10 μm).

The edited *P. trichocarpa* showed slightly improved growth compared with wild-type (Figure 5, A–D). The improved growth was sustained for the period of the study (Figure 5, A–D and Supplemental Figure S11). The morphology, number, size, and wall thickness of the three major stem xylem cell types (fibers, vessels, and rays) are similar between the edited *P. trichocarpa* lines and the wild-type (Supplemental Figure S12). No G-layer could be detected in the lumen of xylem fiber or vessel cells in these plants (Supplemental Figure S12). However, the edited lines developed altered stem wood properties (Table 1) resembling those in TW (i.e., low lignin and high cellulose; Supplemental Table S2, summarized results). The wood of *PtrHSFB3-1-*edited lines (*KO-PtrHSFB3-1*) has less lignin (17.46 ± 0.52% less) and more cellulose (represented by 11.15 ± 0.13% increases in glucose) compared with wild-type (Supplemental Table S2). The alteration was more pronounced in *PtrMYB092* mutants with 26.91 ± 0.69% less lignin and 18.54 ± 0.78% more glucose relative to wild-type (Table 1 and Supplemental Table S2). The mutant results provide strong in vivo validation of the regulatory roles of these TFs in reprogramming cell-wall component biosynthesis during TW formation.

**Table 1.** Wood composition of transgenic and wild-type *P. trichocarpa*

Genotype	Wood sugar content (g/100 g of dry extractive-free wood)										Wood lignin content (g/100 g of dry extractive-free wood)					
	Glucose		Xylose		Galactose		Arabinose		Total carbohydrate		Acid insoluble lignin		Acid soluble lignin		Total lignin	
	Values	Average	Values	Average	Values	Average	Values	Average	Values	Average	Values	Average	Values	Average	Values	Average
WT1	45.75	45.72 ± 0.34	15.24	15.33 ± 0.05	1.42	1.54 ± 0.06	2.42	2.17 ± 0.19	64.83	64.77 ± 0.47	19.76	20.08 ± 0.18	3.66	3.54 ± 0.06	23.42	23.62 ± 0.13
WT2	46.29		15.35		1.60		2.30		65.55		20.38		3.48		23.86	
WT3	45.12		15.41		1.60		1.80		63.93		20.10		3.48		23.58	
*KO-PtrHSFB3-1-5-1*	50.94	50.82 ± 0.06^**^	14.59	14.47 ± 0.37^**^	1.34	1.42 ± 0.06^**^	2.58	2.38 ± 0.13^**^	69.45	69.09 ± 0.3^**^	16.39	16.7 ± 0.18^**^	2.86	2.79 ± 0.09^**^	19.25	19.5 ± 0.12^**^
*KO-PtrHSFB3-1-5-2*	50.77		13.78		1.53		2.42		68.50		16.71		2.91		19.62	
*KO-PtrHSFB3-1-5-2*	50.74		15.04		1.40		2.14		69.32		17.01		2.61		19.62	
*KO-PtrMYB092-15-1*	53.58	54.2 ± 0.35^**^	14.17	14.47 ± 0.15^**^	1.32	1.41 ± 0.05^**^	3.80	3.59 ± 0.11^**^	72.87	73.67 ± 0.46^**^	14.68	14.48 ± 0.11^**^	2.91	2.79 ± 0.06^**^	17.59	17.26 ± 0.16^**^
*KO-PtrMYB092-15-2*	54.80		14.69		1.47		3.51		74.47		14.44		2.69		17.13	
*KO-PtrMYB092-15-3*	54.21		14.53		1.45		3.47		73.67		14.31		2.77		17.07	
*OE-PtrHSFB3-1-4*	43.78	43.76 ± 0.32^*^	14.63	14.72 ± 0.05^**^	1.36	1.48 ± 0.06^**^	2.32	2.09 ± 0.18^**^	62.10	62.04 ± 0.45^**^	22.13	22.53 ± 0.22^**^	3.70	3.39 ± 0.16^**^	25.83	25.92 ± 0.06^**^
*OE-PtrHSFB3-1-7*	44.31		14.74		1.54		2.21		62.79		22.88		3.14		26.02	
*OE-PtrHSFB3-1-10*	43.19		14.79		1.53		1.73		61.24		22.57		3.34		25.91	
*OE-PtrMYB092-6*	43.30	42.29 ± 0.51^**^	15.37	15.34 ± 0.02^**^	1.29	1.31 ± 0.02^**^	1.64	1.57 ± 0.08^**^	61.60	60.52 ± 0.54^**^	24.22	24.57 ± 0.19^**^	3.46	3.35 ± 0.06^**^	27.68	27.91 ± 0.14^**^
*OE-PtrMYB092-7*	41.88		15.35		1.36		1.41		60.00		24.85		3.29		28.15	
*OE-PtrMYB092-9*	41.70		15.29		1.29		1.66		59.95		24.63		3.28		27.91	

* *P *<* *0.05,

** *P *<* *0.01

(Student’s *t* test). ± Numbers indicate one standard error of three biological replicates.

### Overexpressing *PtrHSFB3-1* and *PtrMYB092* negatively affect growth with increased lignin quantity and reduced cellulose content in wood of *P. trichocarpa*

We overexpressed *PtrHSFB3-1* and *PtrMYB092* in *P. trichocarpa* (Figure 5) to further validate the regulatory functions of these TFs. *PtrHSFB3-1* and *PtrMYB092* were individually overexpressed under the control of a CaMV 35S promoter, generating 21 and 8 independent transgenic lines, respectively. We then quantified the transcript abundance of the transgene in SDX and selected three independent lines per TF construct (*OE-PtrHSFB3-1*-*4*, -*7*, -*11*, Figure 5, E;*OE-PtrMYB092*-*6*, -*7*, -*9*, Figure 5, F) with the highest transgene transcript levels. The *PtrHSFB3-1* expression in *OE-PtrHSFB3-1*-*4*, -*7*, and -*11* was 5.73-, 5.5-, and 4.3-fold higher than wild-type, respectively. *OE-PtrMYB092*-*6*, -*7*, and -*9* had *PtrMYB092* transcripts 7.06, 5.51, and 4.93 times more abundant, respectively, than in wild-type.

Overexpression of the two TF genes in transgenics showed reduced plant growth (Figure 5, E–H), an effect opposite to their loss-of-function mutants (Figure 5, A–D). While growth reduction in *OE-PtrHSFB3-1* transgenics was moderate (Figure 5, E, G, and H), overexpression of *PtrMYB092* drastically impaired the growth compared with the wild-type (Figure 5, F–H), and particularly to its knockouts (Figure 5, B–D). *KO-PtrMYB092* mutants grew three- to four-fold higher and developed approximately two times more internodes than *OE-PtrMYB092* transgenics (Figure 5, B–D, F–H). The results suggest an effective role for PtrMYB092 in modulating the growth and development of *P. trichocarpa*. Like in the edited plants, the growth effects were sustained in the overexpressors (Figure 5, A–H and Supplemental Figure S11).

**Figure 6:**
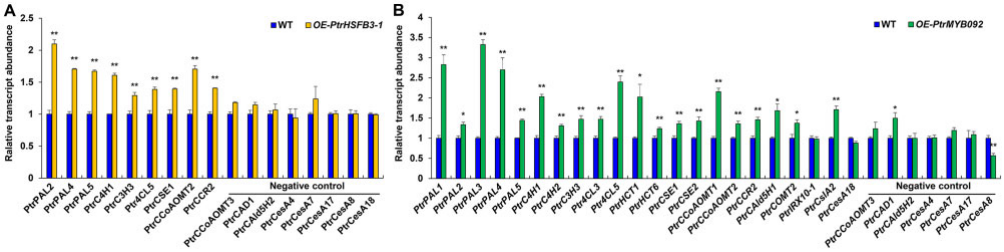
Overexpression of *PtrHSFB3-1* and *PtrMYB092* in *P. trichocarpa* on transcript expression of target cell wall component biosynthetic genes. Relative transcript abundance of cell wall component biosynthetic genes in SDX of transgenic *P. trichocarpa* overexpressing (A) *PtrHSFB3-1* (*OE-PtrHSFB3-1*) and (B) *PtrMYB092* (*OE-PtrMYB092*) compared with wild type (WT). Negative controls denote cell wall component biosynthetic genes not transregulated by PtrHSFB3-1 (A) or PtrMYB092 (B). Error bars represent one SE of three biological replicates (independent transgenic lines). *P < 0.05, **P < 0.01 (Student’s *t* test).

Overexpression of *PtrHSFB3-1* significantly (*P *<* *0.01) transactivated all its direct and indirect cell-wall target genes in SDX of transgenic *OE-PtrHSFB3-1* lines (Figure 6, A). The transcript levels of the eight cell-wall component genes that are not *PtrHSFB3-1*’s targets (negative controls) were not affected (Figure 6, A). Similarly, overexpression of *PtrMYB092* elevated (*P *<* *0.01) the transcript levels of all 11 direct targets and 9 indirect targets (Figure 3, B) in SDX of transgenic *OE-PtrMYB092* lines, while the expression of negative controls remained unaffected (Figure 6, B). The transcript expression of *PtrMYB092*’s other two indirect target genes (*PtrIRX10-1* and *PtrCesA18*) was also not affected in the *OE-PtrMYB092* lines, as was not in the *PtrMYB092* mutants (Figure 4, B), confirming the likely involvement of other unknown regulatory mechanisms. Therefore, in planta expression (mutation in Figure 4 and activation in Figure 6) and the protoplast-derived transregulation analyses (Figure 2) consistently demonstrate that these TFs are transactivators for cell-wall component biosynthesis.

The moderately impeded growth in *OE-PtrHSFB3-1* transgenics did not cause apparent changes in the number and size of xylem cells but increased the wall thickness (Figure 5, I–K). The stunted growth in *OE-PtrMYB092* transgenics also developed significantly less wood areas with smaller and thicker-walled xylem cells per unit area than wild-type plants (Figure 5, I, J, M, and N).

Overexpression of the two TF genes in transgenics showed effects on wood biosynthesis that are also the opposite of their loss-of-function mutants (Figure 5, I–Q and Supplemental Figure S12). For example, *PtrHSFB3-1* overexpression (*OE-PtrHSFB3-1* lines) increased stem wood lignin (9.73 ± 0.24% increase, Table 1 and Supplemental Table S2) and reduced cellulose (4.29 ± 0.71% reduction in glucose, Table 1). *PtrHSFB3-1* knock-outs (*KO-PtrHSFB3-1* lines) had reduced lignin and augmented cellulose (Table 1 and Supplemental Table S2). For *PtrMYB092*, gene overexpression (*OE-PtrMYB092* lines) increased lignin (18.17 ± 0.58% increase, Table 1 and Supplemental Table S2) and gene knockout (*KO-PtrMYB092* lines) reduced lignin (Table 1 and Supplemental Table S2). Similarly, overexpression of *PtrMYB092* reduced cellulose (7.49 ± 1.11% reduction in glucose, Table 1 and Supplemental Table S2) and knockout of *PtrMYB092* increased cellulose (Table 1 and Supplemental Table S2). Alteration of *PtrMYB092* expression had stronger effects on wood component biosynthesis than *PtrHSFB3-1*. Together, the results of strong TW-responsive transrepression and functions (mutation and activation) in transgenics suggest that *PtrHSFB3-1* and *PtrMYB092* are two key TFs in transregulation of cell-wall biosynthesis in TW formation of *P*. *trichocarpa*.

### *PtrHSFB3-1* and *PtrMYB092* transgenic *P. trichocarpa* exhibit altered lignin biosynthesis in TW compared with wild-type

**Figure 7:**
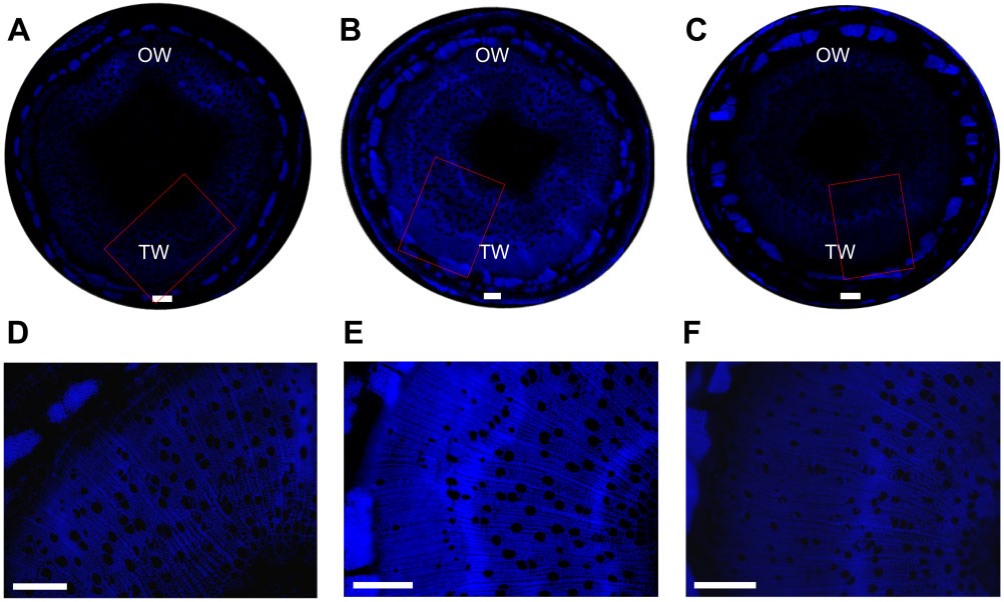
Lignin autofluorescence imaging of *OE-PtrHSFB3-1*, *KO-PtrHSFB3-1*, and wild type under tension stress induced by artificial stem bending for 14 d. Images represent stem cross-sections (9th internode) of wild type (A and D), *OE-PtrHSFB3-1* (B and E), and *KO-PtrHSFB3-1* (C and F). Red rectangles in A–C correspond to magnified images (D–F). Scale bars = 200 μm.

**Figure 8:**
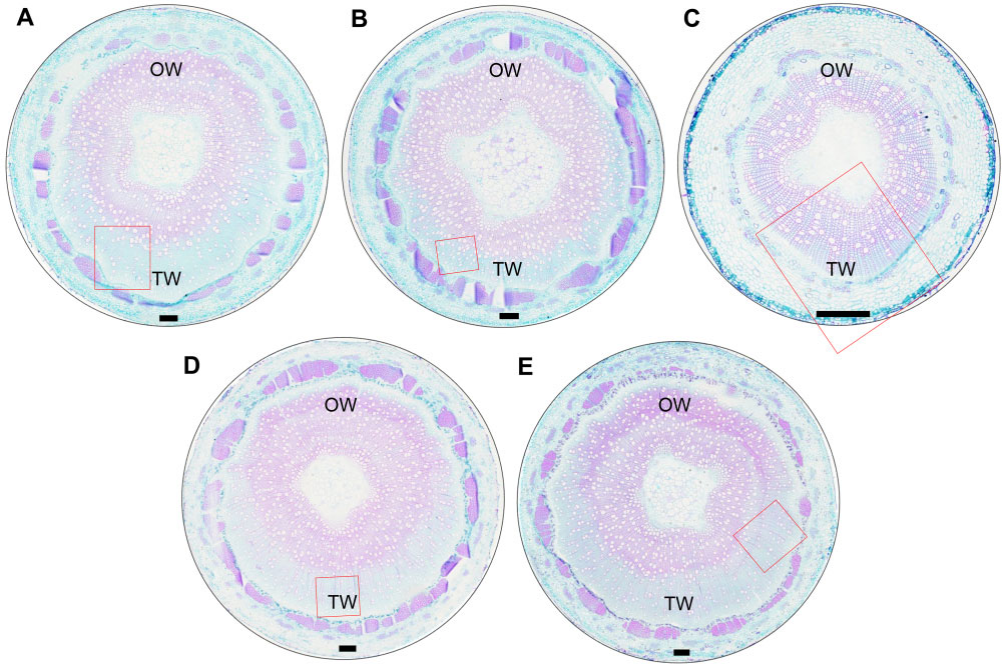
Stem cross-sections of 3-month-old greenhouse-grown transgenic and wild-type *P. trichocarpa* under tension stress induced by artificial stem bending for 21 d. Images represent stem cross-sections (9th internode) of wild type (A), *OE-PtrHSFB3-1* (B), *OE-PtrMYB092* (C), *KO-PtrHSFB3-1* (D), and *KO-PtrMYB092* (E). Cross-sections were stained with safranin *O* and fast green (see the “Materials and methods” section). Red rectangles correspond to magnified images in Supplemental Figure S14. Scale bars = 200 μm.

We next validated the transregulatory roles of *PtrHSFB3-1* and *PtrMYB092* in lignin biosynthesis during TW formation. TW induced by artificial stem bending in *OE-PtrHSFB3-1*, *OE-PtrMYB092*, *KO-PtrHSFB3-1*, *KO-PtrMYB092*, and wild-type *P. trichocarpa* were analyzed by wood composition analysis (Table 2 and Supplemental Table S3), lignin autofluorescence imaging (Figure 7), and staining of stem cross-sections (Figure 8; see the “Materials and methods” section). Lignin exhibits unique autofluorescent properties that can be used to estimate the spatial distribution and content of lignin in xylem tissues based on the relative intensity of the fluorescence (Donaldson, 2013; Decou, 2017). In wild-type *P. trichocarpa*, the intensity of lignin autofluorescence in the TW region was significantly reduced compared with the OW (Figure 7, A and D). The reduced autofluorescence in wild-type TW is consistent with the reduced lignin content (Supplemental Table S1) and transcript abundances of monolignol genes in TW (Figure 1, A).

**Table 2.** TW composition of stem-bending for 21 d in transgenic and wild-type *P. trichocarpa*

Genotype	Wood sugar content (g/100 g of dry extractive-free wood)										Wood lignin content (g/100 g of dry extractive-free wood)					
	Glucose		Xylose		Galactose		Arabinose		Total carbohydrate		Acid insoluble lignin		Acid soluble lignin		Total lignin	
	Values	Average	Values	Average	Values	Average	Values	Average	Values	Average	Values	Average	Values	Average	Values	Average
**B-**WT1	61.63	59.71 ± 1.09	13.74	14.41 ± 0.38	1.80	2.01 ± 0.38	3.61	4.13 ± 0.13	80.79	80.26 ± 32	13.19	13.12 ± 0.03	1.97	2.03 ± 0.03	15.16	15.15 ± 0.02
**B-**WT2	57.85		15.07		2.26		4.52		79.71		13.08		2.08		15.17	
**B-**WT3	59.65		14.40		1.97		4.25		80.28		13.08		2.02		15.1	
**B-** *KO-PtrHSFB3-1-5-1*	65.31	65.11 ± 0.63^**^	12.80	12.78 ± 0.08^**^	1.57	1.72 ± 0.08	3.14	3.45 ± 0.17	82.83	83.07 ± 0.46^**^	10.67	10.65 ± 0.28^**^	2.17	2.10 ± 0.04	12.84	12.75 ± 0.29^**^
**B-** *KO-PtrHSFB3-1-5-2*	66.09		12.62		1.74		3.50		83.95		11.13		2.06		13.20	
**B-** *KO-PtrHSFB3-1-5-2*	63.92		12.91		1.85		3.71		82.42		10.17		2.05		12.22	
**B-** *KO-PtrMYB092-15-1*	64.28	64.73 ± 0.63^*^	13.87	13.66 ± 0.71	2.53	2.78 ± 0.16^*^	5.05	5.30 ± 0.12^*^	85.74	85.96 ± 0.49^**^	9.68	9.34 ± 0.43^**^	1.58	1.73 ± 0.10^*^	11.26	11.07 ± 0.34^**^
**B-** *KO-PtrMYB092-15-2*	66.57		12.19		2.71		5.42		86.90		8.48		1.92		10.42	
**B-** *KO-PtrMYB092-15-3*	63.35		14.91		3.09		5.43		85.23		9.86		1.68		11.54	
**B-** *OE-PtrHSFB3-1-4*	58.88	57.69 ± 0.74	12.94	13.18 ± 0.25^*^	1.37	1.44 ± 0.04^**^	2.74	2.87 ± 0.08^**^	75.93	75.18 ± 0.40^**^	15.29	14.99 ± 0.15^**^	2.17	2.16 ± 0.03^*^	17.46	17.15 ± 0.16^**^
**B-** *OE-PtrHSFB3-1-7*	56.32		13.68		1.51		3.02		74.54		14.87		2.20		17.07	
**B-** *OE-PtrHSFB3-1-10*	57.88		12.92		1.42		2.85		75.08		14.81		2.10		16.92	
**B-** *OE-PtrMYB092-6*	47.60	47.21 ± 0.95^**^	13.15	12.59 ± 0.54^*^	1.39	1.38 ± 0.09^*^	2.79	2.77 ± 0.18^**^	64.93	63.94 ± 0.51^**^	18.25	18.00 ± 0.13^**^	2.16	2.09 ± 0.04	20.41	20.09 ± 0.17^**^
**B-** *OE-PtrMYB092-7*	48.53		11.50		1.22		2.43		63.68		17.95		2.10		20.05	
**B-** *OE-PtrMYB092-9*	45.49		13.12		1.54		3.07		63.21		17.81		2.02		19.82	

* *P *<* *0.05,

** *P *<* *0.01

(Student’s *t* test). ± Numbers indicate one standard error of three biological replicates. **B-** indicate stem-bending for 21d.

*OE-PtrHSFB3-1* (Figure 7, B and E) and *OE-PtrMYB092* (Supplemental Figures S13, B and E) showed uniformly distributed and similarly high levels of lignin autofluorescence in the TW and OW. The fluorescence patterns indicate that overexpression of these TFs is sufficient to prevent the repression of lignin biosynthesis during TW formation. In contrast, *KO-PtrHSFB3-1* (Figure 7, C and F) and *KO-PtrMYB092* (Supplemental Figure S13, C and F) showed reduced lignin autofluorescence in both TW and OW, confirming that lignin biosynthesis is suppressed in the absence of these TFs. The results also suggest that the transactivation of lignin biosynthesis by *PtrHSFB3-1* and *PtrMYB092* is not impaired by tension stress induced by artificial stem bending (Figure 8, A–C). The TW of TF-overexpressed transgenics (Figure 7, B and Supplemental Figure S13, B) exhibited stronger lignin autofluorescence compared with the TW of wild-type and knockout mutants (Figure 7, C and Supplemental Figure S13, C), consistent with the elevated transcript abundance of monolignol genes targeted by *PtrHSFB3-1* and *PtrMYB092* (Figures 2, 3, B, and 5). The regulatory roles of the TFs during TW formation were further validated by quantitative determination of TW composition in the transgenics and wild-type *P. trichocarpa* (Table 2 and Supplemental Table S3). *OE-PtrMYB092* and *OE-PtrHSFB3-1* exhibited significantly higher TW lignin contents of 20.09 ± 0.17 (g/100 g of dry extractive-free wood) and 17.15 ± 0.16, respectively, compared with wild-type (15.15 ± 0.02; Table 2). The knockout mutants showed very low TW lignin contents of 11.07 ± 0.34 (*KO-PtrMYB092*) and 12.75 ± 0.29 (*PtrHSFB3-1*; Table 2). Changes in TW composition were also observed for carbohydrate contents. Glucose increased from 59.71 ± 1.09 in wild-type TW to 65.11 ± 0.63 (*KO-PtrHSFB3-1*) and 64.73 ± 0.63 (*KO-PtrMYB092*) in the knockout mutants (Table 2). *OE-PtrHSFB3-1* and *OE-PtrMYB092* showed reduced glucose contents of 57.69 ± 0.74 and 47.21 ± 0.95, respectively. The glucose content changes are consistent with the staining of cellulose-enriched G-layer in the stem cross-sections of transgenics and wild-type (Figure 8). The TW of TF overexpression transgenics (Figure 8, B and C) showed reduced G-layer staining compared with wild-type (Figure 8, A). In contrast, the TF knockouts (Figure 8, D and E) showed similar staining of G-layer. We did not observe a change in the numbers of fiber and vessel cells between the TW of wild-type (Figure 8, A) and *OE-PtrHSFB3-1*, *OE-PtrMYB092*, *KO-PtrHSFB3-1*, and *KO-PtrMYB092* (Figure 8, B–E and Supplemental Figure S14), confirming that PtrHSFB3-1 and PtrMYB092 regulation during TW formation is specific to monolignol biosynthesis. Taken together, reduced transcript expression of *PtrHSFB3-1* and *PtrMYB092* is necessary to reduce lignin deposition during normal TW formation, and overexpression of *PtrHSFB3-1* or *PtrMYB092* restored lignin biosynthesis in TW.

## Discussion

We demonstrated that TW is formed rapidly under mechanical stress in the stem of greenhouse-grown *P. trichocarpa*, as evidenced by the typical low lignin and high cellulose characteristics (Supplemental Table S1). When TW develops and the TW-specific G-layer emerges in xylem cells (Supplemental Figure S3), the expression of 5 of the 22 monolignol genes was repressed and 3 of the 5 secondary cell-wall CesAs were activated (Figure 1, A). We examined genome-wide transcriptomic response and identified from 61 TW-repressed TFs two most significantly differentially repressed TFs (*PtrHSFB3-1* and *PtrMYB092*) as candidate regulators for altered lignin and cellulose biosynthesis. *PtrHSFB3-1* and *PtrMYB092* are more xylem-specific, and both expressed in xylem fiber and vessel cells (wood forming cells; Figure 1, B). We used protoplasts of wood forming cells coupled with RNA-seq and ChIP to discover the regulatory effects of these two TFs on secondary cell-wall genes. *PtrHSFB3-1* and *PtrMYB092* activate the expression of 31 genes involved in the biosynthesis of lignin, cellulose, and hemicelluloses (Figure 2). CRISPR-based knockout of these two TF genes, which mimics the tension stress-induced repression of the two TFs, resulted in significantly reduced expression of 29 of the 31 target genes (Figure 4). The knockouts developed wood with properties similar to TW (low lignin and high cellulose) during normal growth and under tension stress (Tables 1, 2 and Supplemental Tables S1–S3). The same 29 genes repressed by knockout were all activated in transgenics overexpressing the two TFs (Figure 6), where the transgenic wood had high lignin and low cellulose (Supplemental Table S1 and Table 2). These in vivo validations suggest that *PtrHSFB3-1* and *PtrMYB092* are two key regulators for TW formation. This regulatory knowledge is necessary for advancing genome editing strategies to breed feedstocks with more advantageous traits for materials and energy production. Desirable feedstock traits, such as low lignin and high glucose, as well as improved growth, can now be generated through CRISPR-based editing of *PtrHSFB3-1-* or *PtrMYB092*. These mutants and the overexpressing transgenics generated are unique and valuable varieties for field production to assess their breeding and economic values and to allow understanding of regulations associated with environmental effects on adaptation in species with novel wood and growth traits.

### *PtrHSFB3-1* and *PtrMYB092* directly transregulating cell-wall component genes for TW formation

Plant HSFs play important roles in controlling complex signaling and regulatory systems to cope with heat and other environmental stresses (von Koskull-Doring et al., 2007; Hu et al., 2009; Li et al., 2010; Al-Whaibi, 2011; Scharf et al., 2012; Guan et al., 2013). HSFs confer thermotolerance by inducing the expression of heat shock proteins (HSPs) that chaperone cellular proteins, preventing their misassembling under the heat stress (Payen, 1839; Clos, 1990; Liu et al., 2016). Plant genomes encode multiple HSFs, grouped into three major classes (A, B, and C) based on differences in the protein HR–A/B region (von Koskull-Doring et al., 2007; Scharf et al., 2012; Liu et al., 2018). Functions of many *HSF*s have been demonstrated in Arabidopsis. Typically, they act as central regulators to early heat shock responses (Li et al., 2010; Ikeda et al., 2011; Nishizawa-Yokoi et al., 2011) or disease resistance (Kumar et al., 2009; Ikeda et al., 2011).

There are 28 *HSF*s in *P. trichocarpa* (Hu et al., 2009; Scharf et al., 2012; Liu et al., 2018). Four *HSF*s, two in class A (*PtrHSFA2-2* and *PtrHSFA3-1*) and two in class B (*PtrHSFB3-1* and *PtrHSFB3-2*), are expressed more specifically in SDX (Tunlaya-Anukit, 2014; Shi et al., 2017)*.* While many *HSF*s are associated with heat stress, we discovered a mechanical or tension stress-responsive *HSF*, the PtrHSFB3-1. Transcriptomic profiles of gravistimulation suggested that *PtrHSFB3-1* may be associated with lignin biosynthesis and wood formation in *P. alba* × *P. tremula* (Gerttula et al., 2015). In Arabidopsis, *AtHSFB3* (AT2G41690) shares the closest sequence similarity to *PtrHSFB3-1* and shares a 73% amino acid sequence similarity. The function of AtHSFB3 in Arabidopsis is not clear (Scharf et al., 2012). PtrHSFB3-1 is a direct transactivator of monolignol biosynthetic pathway genes, revealing eight TF–DNA interactions for secondary cell-wall biosynthesis that have not previously been reported.

Reported homologs of *PtrMYB092* include *PtoMYB92* in *P. tomentosa* (Li et al., 2015), and *AtMYB42* and *AtMYB85* in Arabidopsis (Zhong et al., 2008). These MYBs are known activators of a few monolignol biosynthetic pathway genes. Co-expression analysis in *P. alba* × *P. tremula* suggests that *PtrMYB092* may be associated with lignin biosynthesis (Gerttula et al., 2015). We discovered that PtrMYB092 is a direct transactivator of a specific set of monolignol genes (Figure 3, B and C). PtrMYB092 mediates 11 direct TF-monolignol gene interactions.

PtrHSFB3-1 and PtrMYB092 directly transactivate 8 and 11 monolignol pathway genes, respectively, and together they transactivate 14 unique monolignol genes, with five (*PtrPAL4*, *PtrC4H1*, *PtrC3H3*, *PtrCCoAOMT2*, and *PtrCCR2*) being redundant targets of the two TFs (Figure 3). There are at least 23 known monolignol pathway genes in *P. trichocarpa* (Shi et al., 2010; Wang et al., 2018). Suppression of TF-monolignol gene transactivations by repressing either *PtrHSFB3-1* or *PtrMYB092* can effectively reduce lignin formation, but not to the extent of lignin reduction in tension stress (Supplemental Table S1). Under tension stress, both *PtrHSFB3-1* and *PtrMYB092* (and other TFs affecting monolignol genes) are repressed (Supplemental Figure S4 and Supplemental Datasets S1, S2), leading to a more extensive lignin reduction in TW than in transgenics where only one of the two TFs is repressed (Table 1).

### *PtrHSFB3-1* and *PtrMYB092* reprogram transregulation of monolignol genes for lignin biosynthesis in TW that may be distinct from regulation for normal wood formation

*PtrHSFB3-1* and *PtrMYB092* transregulations are highly specific to monolignol biosynthesis because they only mediate direct TF–DNA regulation of 14 unique monolignol genes based on full-transcriptome analysis coupled with ChIP (Figures 2, 3, A and C). In a recent study, we constructed a four-layer TRN mediated by a key TF, *PtrSND1-B1*, for wood formation (Chen et al., 2019). PtrSND1-B1 directs 57 TF_DNA interactions through 17 TFs transregulating 27 cell-wall biosynthesis genes, including 11 monolignol pathway genes. However, bending did not significantly affect the transcript abundance of *PtrSND1-B1*, the master regulator of the four-layer TRN. The TRN’s 17 TF genes do not include *PtrHSFB3-1* and *PtrMYB092*. These 17 TFs were screened based on a genome-wide approach to be comprehensive for PtrSND1-B1-mediated wood formation. Whether any of these 17 TFs are the direct transregulators of *PtrHSFB3-1* or *PtrMYB092* is unknown in *P. trichocarpa* or any *Populus* species.

These results suggest that TW formation may be triggered by reprogrammed transregulation of cell-wall genes mediated by a tension-induced network (or TW-TRN), where TFs like *PtrHSFB3-1* and *PtrMYB092* are involved. The *PtrHSFB3-1* and *PtrMYB092* containing TW-TRN may also encompass associated TFs that can directly transregulate cellulose and hemicellulose biosynthetic genes more specifically for TW formation. This is because PtrMYB092 can significantly transactivate indirectly a major secondary cell-wall cellulose synthase gene, *PtrCesA18*, and two hemicellulose synthases, *PtrIRX10-1* and *PtrCslA2* (Figures 2, B and 3, C). The indirect transregulation must be mediated by *PtrMYB092* through intermediate TFs, which in turn transregulate directly (or indirectly) these polysaccharide biosynthetic genes. The activation of *PtrCesA18* and *PtrIRX10-1* and *PtrCslA2* may be associated more with the secondary cell-wall biosynthesis instead of the G-layer, as suggested by results from bending and transgenic experiments (Supplemental Table S1 and Table 1).

### G-layer formation may be mediated by transactivation of cellulose synthase genes but not by *PtrHSFB3-1* and *PtrMYB092*

An important characteristic of TW is a G-layer in the lumen of fiber cells (Supplemental Figures S2, S3) and the G-layer has essentially no lignin (Kaku et al., 2009; Gorshkova et al., 2015). Because knockout of *PtrHSFB3-1* and *PtrMYB092* mimics TW formation, we examined the stem cross-sections of *PtrHSFB3-1* and *PtrMYB092* mutant lines but could not detect any G-layer (Supplemental Figure S12, A–D). Therefore, *PtrHSFB3-1* and *PtrMYB092* cannot mediate G-layer biosynthesis. Glucose content was elevated in the TW of wild-type, *KO-PtrHSFB3-1*, and *KO-PtrMYB092* lines, but reduced in the TW of *OE-PtrHSFB3-1* and *OE-PtrMYB092* lines (Tables 1, 2 and Supplemental Tables S1–S3). The results suggest that cellulose biosynthesis for G-layer formation in TW is not directly mediated by either *PtrHSFB3-1* or *PtrMYB092*. Tension stress represses the expression of *PtrHSFB3-1* and *PtrMYB092* in secondary cell walls leading to reduced lignin, which may be compensated by high cellulose in these walls as we have previously observed (Hu et al., 1999; Li et al., 2003; Novaes et al., 2010; Lu et al., 2013). Therefore, we suggest that changes in TW’s chemical composition are due to increased cellulose in the affected xylem secondary cell walls and the G-layer in fiber lumen, and to the reduced lignin biosynthesis mediated at least by *PtrHSFB3-1* and *PtrMYB092* in secondary cell walls.

### *PtrHSFB3-1* and *PtrMYB092* are possible repressors for growth and development in *P. trichocarpa*

In this study, we also discovered roles for *PtrHSFB3-1* and *PtrMYB092* in regulating plant growth (Figure 5). Plant height and number of stem internodes were adversely affected by the transcript levels of *PtrHSFB3-1* and *PtrMYB092*. The effects were particularly prominent for *PtrMYB092* (Figure 6, F–H). Because essentially every aspect of plant growth and development is regulated by phytohormones (Vanstraelen and Benková, 2012), such as auxin/IAA or gibberellins (Vargas et al., 2016), such regulations may be affected in transgenics where expression of *PtrMYB092* is constitutively perturbed. Perhaps, PtrMYB092 can interact with a receptor protein perceived with phytohormones to interrupt a transduction pathway where a specific amount of the hormone can be released to maintain normal plant growth and development. High levels of *PtrMYB092* may inhibit the hormone’s release, resulting in impeded growth, whereas the absence of *PtrMYB092* may achieve the opposite. The *PtrHSFB3-1* and *PtrMYB092* mutants and overexpressing transgenics produced here are unique systems for understanding the interactions between TRNs and hormonal pathways/networks that coordinate regulations of metabolisms, growth, and adaptation in plants, particularly in forest tree species.

## Materials and methods

### Plant materials

*Populus trichocarpa* (genotype Nisqually-1) plants were grown and propagated in an indoor greenhouse. The temperature was maintained at ∼23°C with 16-h light/8-h dark photoperiod (light intensity ∼300 μE m^−2^ s^−1^), and 60%–80% humidity, as previously described (Li et al., 2011). Greenhouse plants were watered daily to soil saturation. Clonal plants were propagated by lateral branches (∼15 cm) rooted in water. The rooted branches were planted in 16-cm diameter pots filled with a soil mixture of peat moss and Metro-Mix 200 (2:1 ratio, respectively). Six-month-old healthy plants were used for RNA extraction and SDX protoplast isolation.

### Artificial stem bending to induce TW formation

*Populus trichocarpa* trees (6 months old) were used to induce TW formation by mechanical bending of the stem to an angle of 90° (Supplemental Figure S1). SDX tissues were collected 3 d and 7 d after bending (Supplemental Figure S1). SDX was lightly scraped (0.2–0.5 mm in depth) using a razor blade from the upper (TW region) of the bent stems, as well as from corresponding locations on the controls (non-bent trees). SDX tissues were immediately frozen in liquid nitrogen for transcriptomic analysis.

### Total RNA extraction

Total RNA from *P. trichocarpa* was isolated using the Qiagen RNeasy Mini kit (Invitrogen) for RNA-seq, cDNA cloning, and RT-qPCR. For transcriptomic analysis, the quality of isolated RNA was determined using a Bioanalyzer 2100 (Agilent), and RNA samples with RIN value greater than 9.0 were used for RNA-seq following Lin et al. (2013) for the artificially bent stem samples and non-bent controls (GEO accession number: GSE153793).

### Gene cloning

The full-length coding sequences of *PtrHSFB3-1* and *PtrMYB092* were amplified from 20 ng/µL of cDNA reverse transcribed using the SuperScript III kit (Invitrogen) from total RNA of *P. trichocarpa* SDX. Primers for gene cloning are listed in Supplemental Dataset S8. The amplified coding sequences were integrated into the *pENTR/D-TOPO* vector (Invitrogen) and verified by Sanger sequencing.

### Constructs for transient overexpression of PtrHSFB3-1 and PtrMYB092 in SDX protoplasts

The coding sequences of *PtrHSFB3-1* and *PtrMYB092* were cloned from the *pENTR-D-TOPO* vector into *pUC19-35S-RfA-35S-sGFP* (Li et al., 2012) using LR reactions, generating transient overexpression constructs *pUC19-35S-PtrHSFB3-1-35S-sGFP* and *pUC19-35S-PtrMYB092-35S-sGFP*.

### Histochemical and histomorphological analyses

Stem segments (9th internode) from 0-, 3-, 7-, 14-, and 21-d stem bending trees and *KO-PtrHSFB3-1*, *KO-PtrMYB092*, *OE-PtrHSFB3-1*, and *OE-PtrMYB092* transgenic and wild-type plants of 180 d old were harvested. Paraffin-embedded stem sections were prepared as previously described (Li et al., 2019). Cross-sections (16-µm thick) were stained with safranin O and fast green for microscopy (Leica DM6B) analysis to characterize the formation of TW by the deposition of G‐layers, changes in the intensity of lignin staining, and changes in vessel cell numbers.

### Quantitative PCR

Quantitative PCR was carried out using the Mx3000P Real-Time PCR System (Agilent). FastStart Universal SYBR Green Master Mix (Roche) was used for the amplification and detection of target genomic DNA (qPCR) or cDNA (RT-qPCR). cDNAs were synthesized by reverse transcription using the SuperScript III Reverse Transcriptase (Invitrogen) following manufacturer’s procedures. Transcript level was normalized to the expression of 18S rRNA (Schmittgen and Livak, 2008). The target DNA enrichment level was normalized to the expression of *PtrACTIN* (Li et al., 2019). Three technical replicates were carried out for each quantitative PCR assay. Primers for gene cloning are listed in Supplemental Dataset S8.

### Constructs for ChIP assays

Restriction sites for *BamH* I and *Xho* I were added to the 5′ and 3′ of the coding sequences of *PtrHSFB3-1* and *PtrMYB092*, respectively, using primers listed in Supplemental Dataset S8. The PCR amplified coding sequences were then restriction digested and ligated to generate the transient expression vectors (*pUC19-35S-PtrHSFB3-1-sGFP* and *pUC19-35S-PtrMYB092-sGFP*).

### Subcellular localization of *PtrHSFB3-1* and *PtrMYB092*

*pUC19-35S-PtrHSFB3-1-sGFP* and *pUC19-35S-PtrMYB092-sGFP* plasmids were purified using the CsCl density-gradient ultracentrifugation method (Lin et al., 2014) and the concentration was diluted to 2 µg/µL. SDX protoplasts (1 × 10^6^ cells) were isolated, transfected, and cultured as described previously (Lin et al., 2013, 2014), with mannitol concentration in buffers MMG and WI reduced from 0.5 to 0.25 M. *pUC19-35S-PtrHSFB3-1-sGFP* and *pUC19-35S-PtrMYB092-sGFP* plasmids were individually co-transfected with *pUC19-35S-H2A*-cherry (a nuclear localization marker) into SDX protoplasts for the subcellular localization of the two TFs. After 7 h incubation, nuclear localization was observed by fluorescence imaging using an LSM 710 fluorescence laser scanning microscope (Zeiss). The excitation and emission wavelengths of GFP were 488 and 492–543 nm, respectively, and mCherry was detected using 561 (excitation) and 582–662 nm (emission).

### Gene expression analysis in SDX protoplasts

*pUC19-35S-PtrHSFB3-1-35S-sGFP* and *pUC19-35S-PtrMYB092-35S-sGFP* plasmids were prepared and individually transfected into SDX protoplasts as described above. After culturing for 12 h, protoplasts (5–8 × 10^5^ cells) were collected by centrifugation at 600 × *g* for 5 min at room temperature. Total RNA was isolated from the SDX protoplasts using the RNeasy Mini kit (Invitrogen) as described previously (Chen et al., 2019). RT-qPCR analyses were carried out as described above. Three biological replicates for each transfection were performed. Primers for RT-qPCR are listed in Supplemental Dataset S8.

### Transcriptomic analysis of TF overexpression in SDX protoplasts and identification of DEGs

The quality and concentration of total RNA (>1 μg) isolated from SDX protoplasts overexpressing *PtrMYB092*, *PtrHSFB3-1*, and sGFP individually were verified using a 2100 Bioanalyzer (Agilent). Total RNA (1 μg) for each sample was used for library construction using the TruSeq RNA library preparation kit (Illumina). A total of nine libraries (three biological replicates) were sequenced using HiSeq 2500/4000 (Illumina). The sequencing of libraries was performed by Beijing Genomics Institute (BGI; GEO accession number: GSE154167). Sequencing reads were mapped to *P. trichocarpa* genome v3.0 (Phytozome, www.phytozome.com) using Bowtie 2 (version 2.1.0; Langmead and Salzberg, 2012). DEGs (false discovery rate, FDR < 0.05; fold-change, FC > 2 or < 0.8) induced by the overexpression of *PtrMYB092* and *PtrHSFB3-1* were identified by comparing (Robinson et al., 2010) to SDX protoplasts overexpressing sGFP (control).

### ChIP RT-qPCR

ChIP analysis using SDX protoplasts was performed according to our previously established procedure (Chen et al., 2019; Yeh et al., 2019). DNA plasmids (∼3 mg) of *pUC19-35S-PtrMYB092-GFP*, *pUC19-35S-PtrHSFB3-1-sGFP*, and *pUC19-35S-sGFP* (control) constructs were individually transfected into SDX protoplasts (1 × 10^7^ cells). After incubation for 12 h in the dark at room temperature, the protoplasts were collected in 50-mL tubes by centrifugation at 750 × *g* for 10 min and re-suspended in 20 mL of WI buffer (0.2 M MES pH 5.7, 0.8 M mannitol, 2 M KCl), then 540 μL of formaldehyde was added, mixed well, and incubated for 10 min for cross-linking at room temperature. The cross-linked protoplasts were washed twice using cold WI buffer and lysed using lysis buffer (50 mM Tris–HCl pH 5.7, 10 mM EDTA, 1% (w/v) SDS, 1 mM PMSF, 1 µg/mL pepstatin A, 1 µg/mL leupeptin) for nucleus purification and chromatin extraction. Chromatin was fragmented using a Bioruptor (Diagenode) for three rounds of five cycles at maximum power. The average size of the DNA-protein fragments was ∼0.75 kb. The chromatin fragments were immunoprecipitated using 5 μg of anti-GFP antibody (Abcam, ab290; Li et al., 2019). DNA from the immunoprecipitants was purified using the MinElute kit (Qiagen) and analyzed by ChIP qPCR as previously described (Mukhopadhyay et al., 2008; Li et al., 2014). Three biological replicates were carried out for each ChIP qPCR experiment. Primers for ChIP qPCR are shown in Supplemental Dataset S8.

### Generation of transgenic *P. trichocarpa*

The coding sequences of *PtrHSFB3-1* and *PtrMYB092* were amplified using primers with *BamH* I and *Sac* I restriction endonuclease cut sites. The PCR products were restriction digested by *BamH* I and *Sac* I and inserted in *pBI121* vector digested with *BamH* I and *Sac* I. The assembled transformation plasmids were then sequence-verified and transformed into *A. tumefaciens* strain GV3101 for *Agrobacterium*-mediated transformation of *P. trichocarpa*, following our established procedures (Song et al., 2006).

The transcript abundances of *PtrHSFB3-1* and *PtrMYB092* in SDX of transgenic plants were quantified by RT-qPCR, as described above. The transgenic *P. trichocarpa* lines with the highest transgene expression levels for *PtrHSFB3-1* and *PtrMYB092* genes were selected and maintained in a greenhouse for phenotypic analyses.

### CRISPR-based editing of *PtrHSFB3-1* and *PtrMYB092* in transgenic *P. trichocarpa*

We used CRISPR-Cas9 (Ueta et al., 2017) to individually target the loss-of-function editing of *PtrHSFB3-1* and *PtrMYB092* in transgenic *P. trichocarpa*. Guide RNAs (sgRNA) targeting the coding sequences of *PtrMYB092* and *PtrHSFB3-1* were designed using the software CRISPR-P2.0 (http://crispr.hzau.edu.cn/cgi-bin/CRISPR2/CRISPR). sgRNAs were verified using IGV (http://software.broadinstitute.org/software/igv) to be free of single-nucleotide polymorphisms (SNPs) that may interfere with target DNA editing. sgRNA sequences were restriction digested using *Bsa* I and inserted into the transformation vector *pEgP237-2A-GFP* digested with *Bsa* I (Ueta et al., 2017). The assembled transformation plasmids were then transformed into *A. tumefaciens* strain GV3101 for *Agrobacterium*-mediated transformation of *P. trichocarpa* following our established procedures (Song et al., 2006).

CRISPR RNP in vitro cleavage assays were used to verify the specificity of sgRNAs for editing target DNA. Assembly PCR reactions using primers (Supplemental Dataset S8) were used to produce the double-stranded DNA templates for sgRNAs production. T7 Quick High Yield RNA Synthesis Kit (NEB) was used to carry out the gRNA in vitro transcription. Recombinant SpCas9 protein was prepared following (Liang et al., 2017). In vitro cleavage assays were performed by mixing recombinant SpCas9 with sgRNA and linearized DNA target following established procedures (Liang et al., 2017).

To detect target gene editing in the transgenic *P. trichocarpa*, primers flanking the sgRNA target sequence were used to carry out PCR amplification of isolated genomic DNA. PCR products (500–700 bp) were inserted into the *pMD18-T* cloning vector, and 20 positive colonies were sequenced. For each transgene construct, three independent CRISPR transgenic lines were selected to characterize target gene editing and the downstream transcriptional response using RT-qPCR.

### Artificial stem bending to induce TW formation in transgenic and wild-type *P. trichocarpa*

Three-months-old transgenic and wild-type *P. trichocarpa* were used to induce TW formation by mechanical bending of stem segments (9th internode) to an angle of 90° (Supplemental Figure S1). Stem segments (9th internode) from bent stems of transgenic *KO-PtrHSFB3-1*, *KO-PtrMYB092*, *OE-PtrHSFB3-1*, *OE-PtrMYB092*, and wild-type controls were harvested for imaging of lignin autofluorescence (14-d bending) and stem cross-sections stained using safranin O and fast green (21-d bending).

### Scanning electron microscopy

The 9th internode stem segments of *KO-PtrHSFB3-1*, *KO-PtrMYB092*, *OE-PtrHSFB3-1*, and *OE-PtrMYB092* transgenics and wild-type *P. trichocarpa* were cut transversely into 0.1 mm segments using a razor blade. The segments coated with gold powder (60 s at 10 mA) were imaged using a Nanotech JCM-5000 scanning electron microscopy at a high vacuum (15 kV).

### Wood composition analysis

Stem wood from unbent and 21-d-bent transgenic and wild-type trees were collected for wood composition analysis. The stem segments of 10–18 internodes were collected for *KO-PtrHSFB3*, *KO-PtrMYB092*, and *OE-PtrHSFB3-1* plants. Due to the reduced growth of *OE-PtrMYB092* plants, stem wood from 10 to 23 internodes was collected. The stem samples were cut into 10-cm segments, and for the bent stem samples, the TW region was carefully excised using a razor blade for wood composition analysis. Treatment for extractive-free wood and quantification of wood lignin and sugar contents were carried out following established procedures (Lu et al., 2013; Wang et al., 2018).

### Lignin autofluorescence imaging of stem cross-sections in bent stems of *OE-PtrHSFB3-1* and *OE-PtrMYB092*

Paraffin-embedded stem sections (9th internode) were prepared as previously described (Li et al., 2019). Cross-sections (16-µm thick) were dewaxed with xylene and ethanol for UV microscopy (Leica DM6B) analysis to detect the lignin content change in bending stems. The excitation and emission wavelengths in the detection of lignin autofluorescence were 350 and 435–485 nm, respectively. Identical excitation light intensity was used for all samples.

## Accession numbers

Sequence data from this article can be found in the GenBank/EMBL data libraries under accession numbers GSE153793 and GSE154167. Refer to Supplemental Datasets for gene names and identifiers mentioned in this paper.

## Supplemental data

**Supplemental Figure S1.** Artificial stem bending to induce TW formation in *Populus trichocarpa.*

**Supplemental Figure S2.** Stem cross-section of *P. trichocarpa* TW formation.

**Supplemental Figure S3.** Time-course stem bending to induce TW formation.

**Supplemental Figure S4**. The transcript abundance of *PtrHSFB3-1* and *PtrMYB092* in TW of *P. trichocarpa.*

**Supplemental Figure S5.** RT-qPCR of *P. trichocarpa* SDX protoplasts overexpressing *PtrHSFB3-1*, *PtrMYB092*, or *GFP* control.

**Supplemental Figure S6.** Quality of isolated total RNA from transfected SDX protoplasts of *P. trichocarpa*.

**Supplemental Figure S7.** The transcriptional regulation of the GFP-tagged and untagged *PtrHSFB3-1* and *PtrMYB092*.

**Supplemental Figure S8.** sgRNA target sequence for editing *PtrHSFB3-1* and *PtrMYB092*.

**Supplemental Figure S9.** Validation of sgRNAs using CRISPR RNP in vitro cleavage assays.

**Supplemental Figure S10.** CRISPR-based editing of *PtrHSFB3-1* and *PtrMYB092* in *P. trichocarpa.*

**Supplemental Figure S11.** Plant growth and number of stem internodes in transgenic *P. trichocarpa*.

**Supplemental Figure S12.** Cell wall thickness in xylem of transgenic *P. trichocarpa*.

**Supplemental Figure S13.** Lignin autofluorescence imaging of *OE-PtrMYB092*, *KO-PtrMYB092* transgenics, and wild-type under tension stress induced by artificial stem bending.

**Supplemental Figure S14.** Stem cross-section of TW formation in wild-type, transgenic, and mutant *P. trichocarpa*.

**Supplemental Table S1.** Wood composition of vertical stem (WT) and stem-bending for 21 d (WTB) in wild-type *P. trichocarpa*.

**Supplemental Table S2.** Percentage changes in wood composition of transgenics *P. trichocarpa* relative to wild type.

**Supplemental Table S3.** Percentage changes in TW composition of transgenic *P. trichocarpa* relative to wild type.

**Supplemental Dataset S1.** DEGs in TW after 3-d stem bending.

**Supplemental Dataset S2.** DEGs in TW after 7-d stem bending.

**Supplemental Dataset S3.** DEGs in protoplasts overexpressing *PtrHSFB3*-*1*.

**Supplemental Dataset S4.** DEGs in protoplasts overexpressing *PtrMYB092*.

**Supplemental Dataset S5.** Significant GO of DEGs transregulated by *PtrHSFB3-1*.

**Supplemental Dataset S6.** Significant GO of DEGs transregulated by *PtrMYB092*.

**Supplemental Dataset S7.** Gene ID of *PtrHSFB3-1*, *PtrMYB092*, and their DEGs in secondary cell wall biosynthesis.

**Supplemental Dataset S8.** Primer sequences used in this paper.

## Funding

This work was supported by the National Key Research and Development Program of China (2016YFD0600106), the National Natural Science Foundation of China (Grant 31430093, 31670674), and the Innovation Project of State Key Laboratory of Tree Genetics and Breeding (Northeast Forestry University, grant number A01). This work was also financially support by the Fundamental Research Funds of the Central Universities of China grant 2572018CL01 and Heilongjiang Touyan Innovation Team Program.

*Conflict of interest statement.* None declared. 

## Supplementary Material

kiab038_Supplementary_Data

## Notes

J.P.W., V.L.C., W.L., and B.L. designed the research. B.L., J.L., J.Y., Z.W., and Y.S. performed the research. B.L., J.P.W., Y.-C.J.L., and V.L.C. analyzed the data. B.L., J.P.W., V.L.C., and W.L. wrote the article with input from all co-authors.

The author responsible for distribution of materials integral to the findings presented in this article in accordance with the policy described in the Instructions for Authors (https://academic.oup.com/plphys/pages/general-instructions) is: Jack P. Wang (jpwang@ncsu.edu).
