# Supplementary material for: Transcriptional reprogramming of xylem cell wall biosynthesis in tension wood
Source: Plant Physiol. 2021 Feb 2;186(1):250–69. doi: 10.1093/plphys/kiab038 (PMC8154086; doi:10.1093/plphys/kiab038)
Supplement: kiab038_Supplementary_Data [file kiab038_supplementary_data.zip › pp2020-ra-01016d supplemental data.pdf]

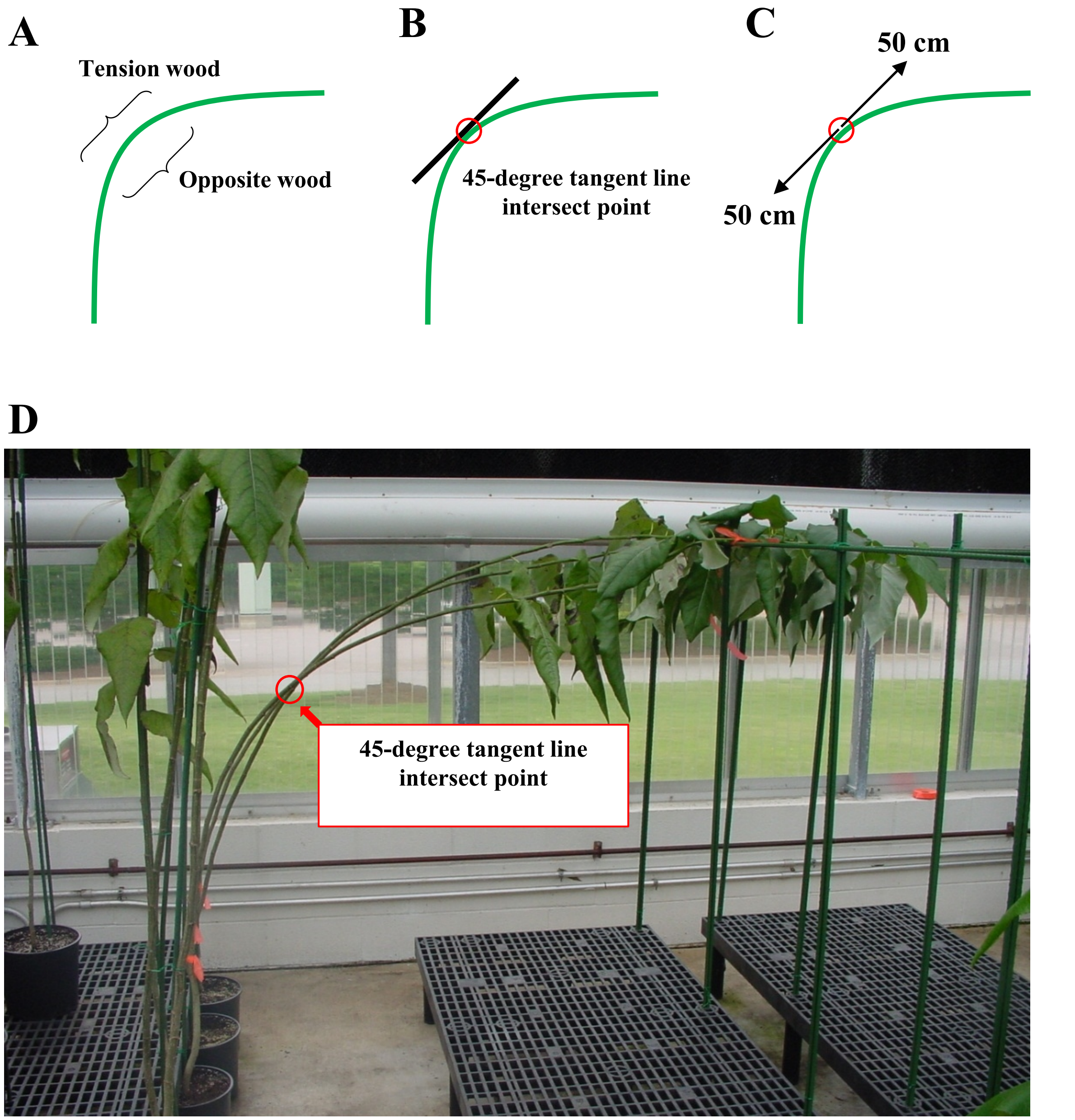

Supplemental Figure S1. Artificial stem bending to induce tension wood formation in *P. trichocarpa.*

(**A-C**) Schematic diagram of stem bending and collected sampled location. (**D**) Tension wood formatting of 6-month-old *P. trichocarpa* in greenhouse.

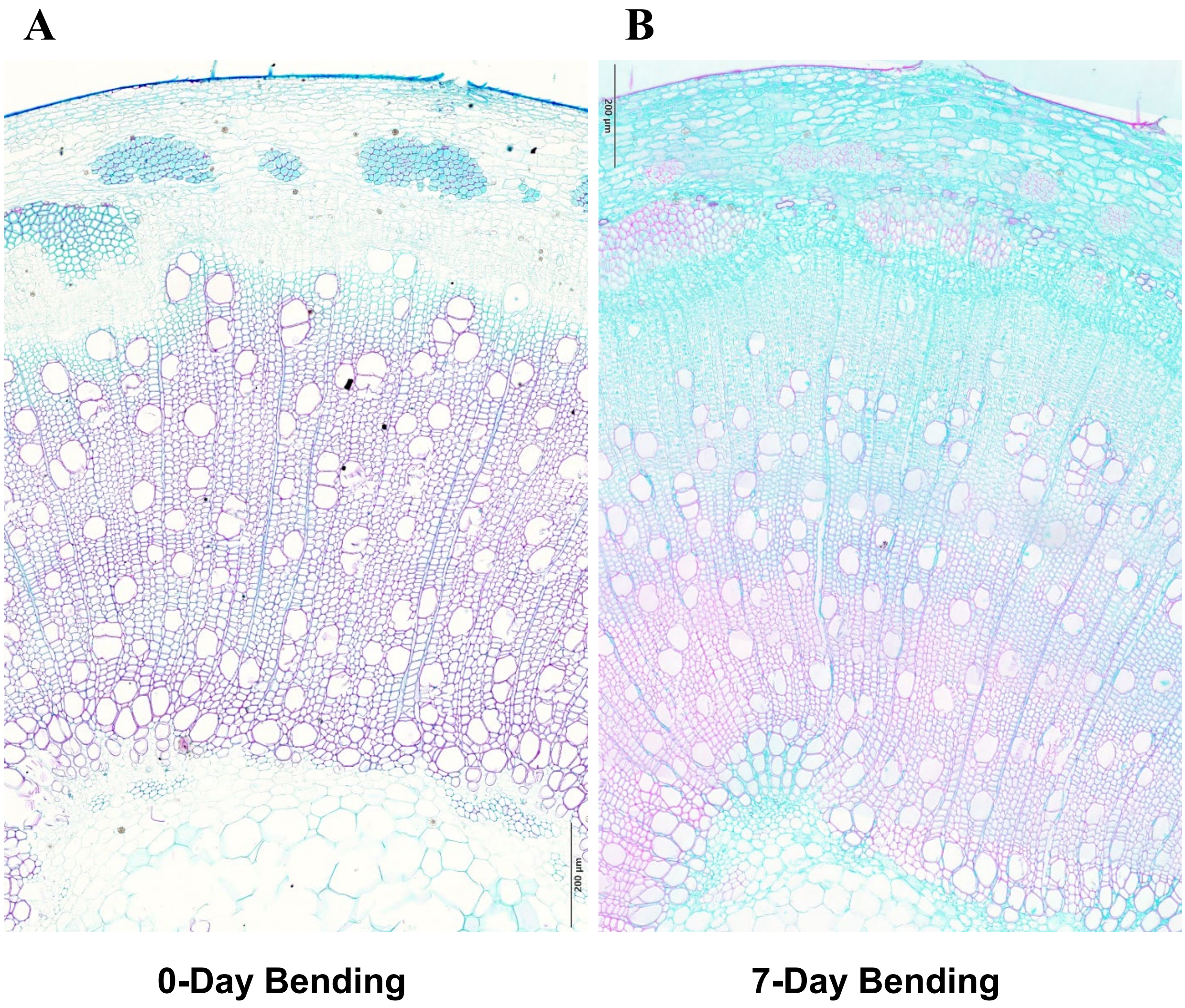

Supplemental Figure S2. Stem cross-section of *P. trichocarpa* tension-wood formation.

Stem cross-section of 6-month-old greenhouse grown *P. trichocarpa* 0 day (**A**) and 7 days (**B**) after artificial bending to induce tension wood formation. Cross sections were stained with safranin O and fast green.

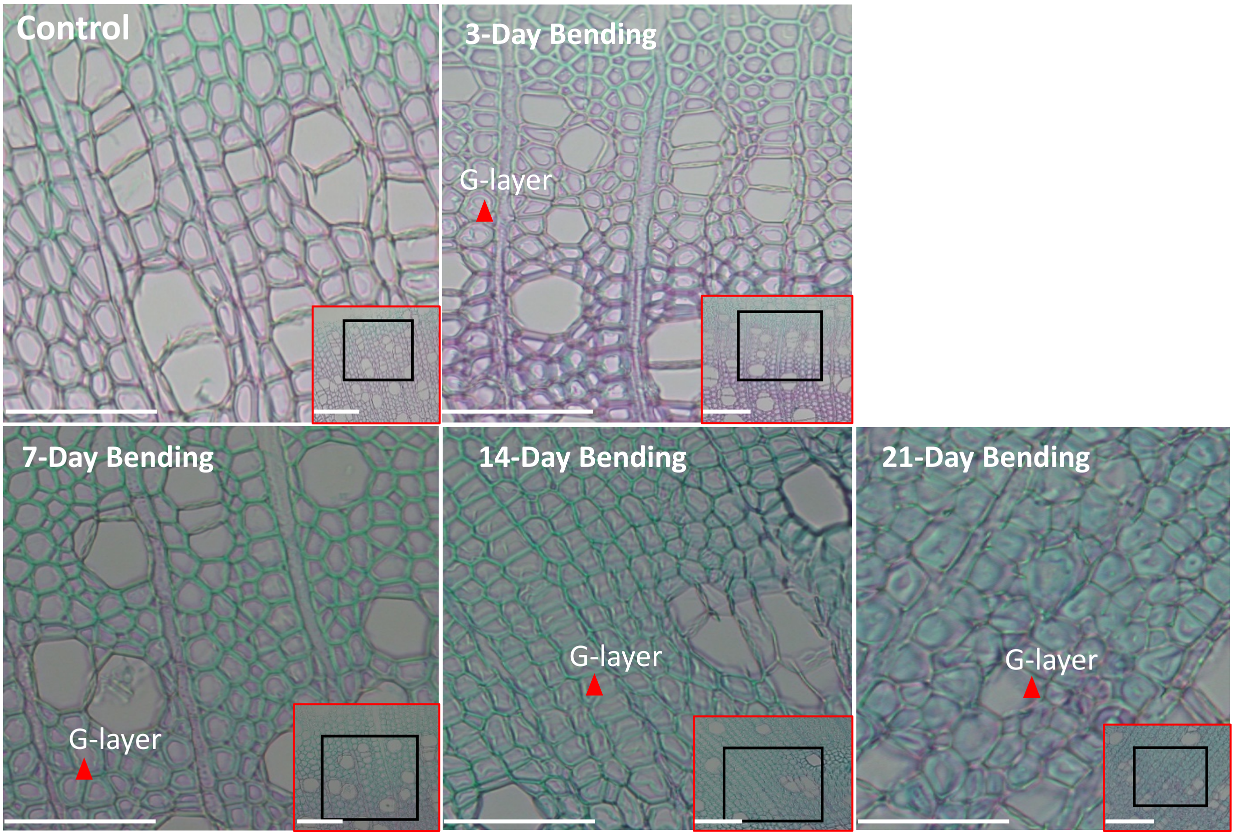

Supplemental Figure S3. Time-course stem bending to induce tension-wood formation.

Time-course induction of TW formation 0, 3, 7, 14, and 21 days after stem bending for 6-month-old greenhouse grown *P. trichocarpa*. TW formation is evident 3 days after bending and becomes prominent 7 days after bending. Red arrows represent the formation of G-layers. Cross sections were stained with safranin O and fast green. Black boxes indicate the region that was amplified. Bars=100 μm.

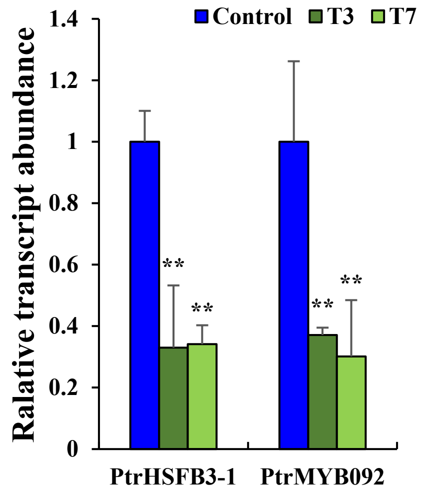

Supplemental Figure S4. The transcript abundance of *PtrHSFB3-1* and *PtrMYB092* in tension wood of *P. trichocarpa.*

Relative transcript abundance (fold change) of PtrHSFB3-1 and PtrMYB092 in SDX of upright stem (control), 3-day stem bending (T3) and 7-day stem bending (T7). **$p < 0.01$ (Student's t-test). Error bars indicate one standard error of three biological replicates.

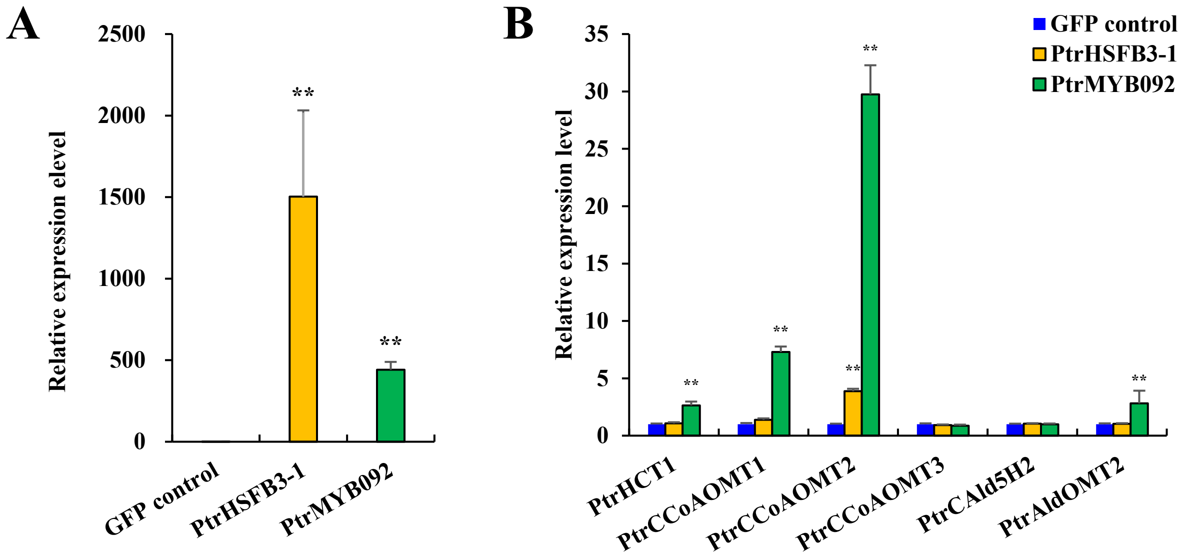

Supplemental Figure S5. RT-qPCR of *P. trichocarpa* SDX protoplasts overexpressing *PtrHSFB3-1*, *PtrMYB092*, or *GFP* control.

(A) RT-qPCR quantification of transcript abundance (fold change) of transgenes in SDX protoplasts overexpressing GFP (control, set to 1), *PtrHSFB3-1*, and *PtrMYB092*. Error bars indicate one SE of three biological replicates from independent PEG transfected protoplasts. Asterisks indicate significant difference between control (GFP) and transfected samples with *PtrMYB092*, *PtrHSFB3-1* (***p* < 0.01, Student's t-test). **(B)** RT-qPCR detection of the expression level of *PtrCAld5H2*, *PtrAldOMT2*, *PtrHCT1*, *PtrCCoAOMT1*, *PtrCCoAOMT2* and *PtrCCoAOMT3* in the protoplasts of *P. trichocarpa* SDX transfected with *PtrMYB092*, *PtrHSFB3-1* and GFP. Error bars indicate one SE of three biological replicates from independent PEG transfected experiments. Asterisks indicate significant difference between control (GFP) and transfected samples with *PtrMYB092 and PtrHSFB3-1* (***p* < 0.01, Student's t-test).

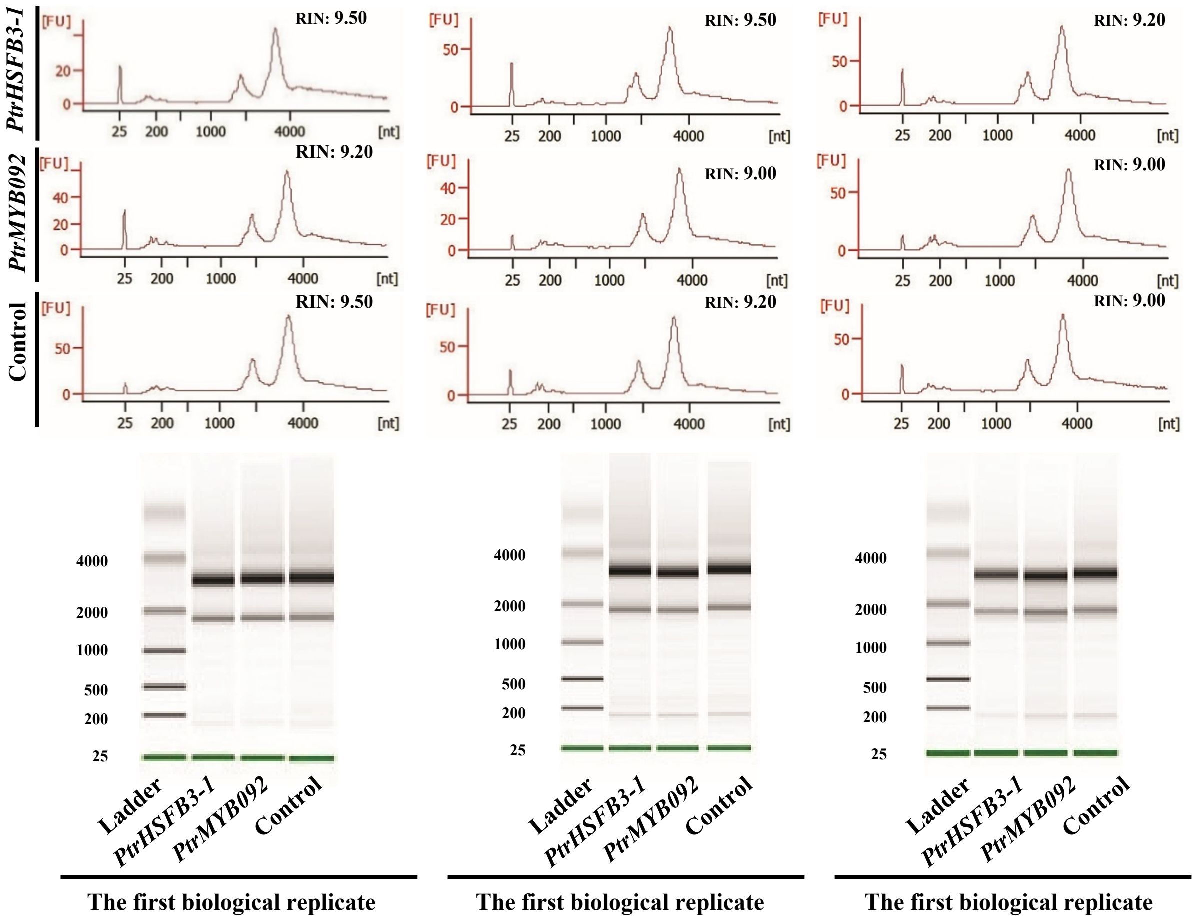

Supplemental Figure S6. Quality of isolated total RNA from transfected SDX protoplasts of *P. trichocarpa.*

Evaluation of total RNA quality extracted from SDX protoplasts of *P. trichocarpa* transfected with *PtrMYB092*, *PtrHSFB3-1* and GFP by a Bioanalyzer 2100 (Agilent). RIN values in electropherogram indicate the integrity total RNA, The first peak and the second peak between 1000 and 4000 are 18s and 28s, respectively. All RIN ≥ 9.00.

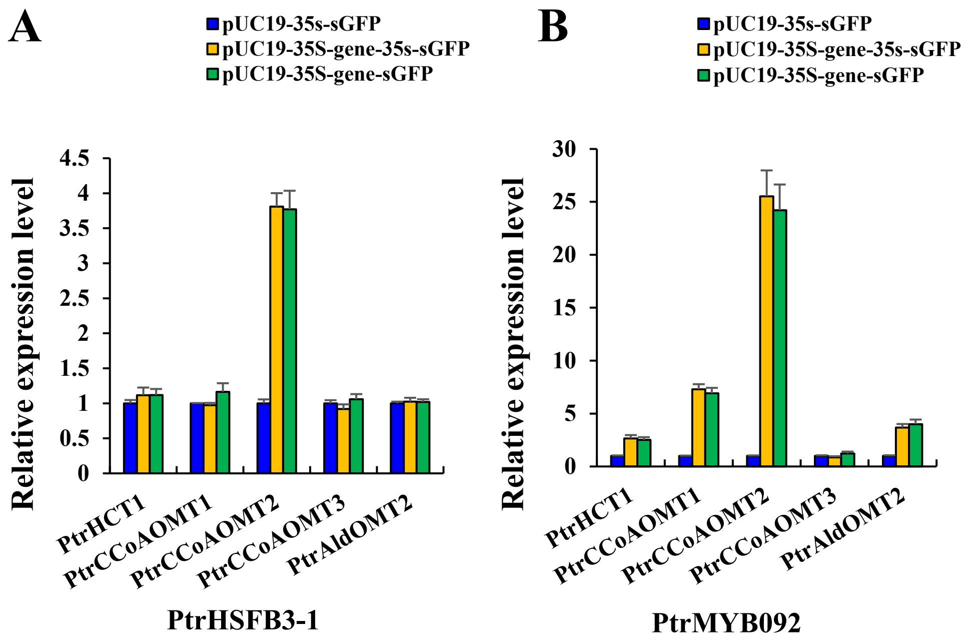

Supplemental Figure S7. The transcriptional regulation of the GFP-tagged and untagged *PtrMYB092* and *PtrHSFB3-1*.

RT-qPCR analysis showed that *PtrHSFB3-1* and *PtrMYB092* with and without GFP tag had similar levels in regulating the expression of randomly selected monolignol genes. (A) *PtrHSFB3-1* (B) *PtrMYB092*. Error bars represent one standard error (SE) of three biological replicates. The verified GFP-tagged TFs were used in ChIP assays.

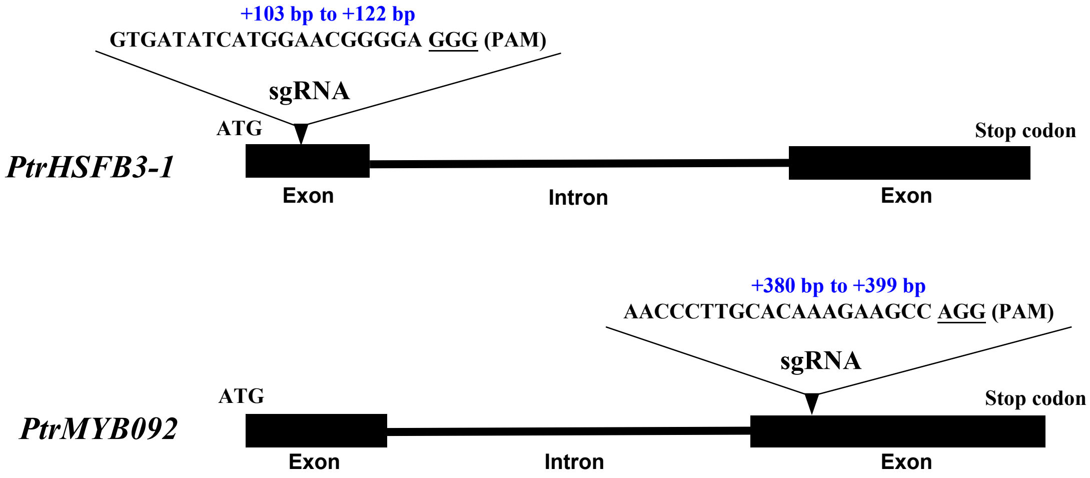

Supplemental Figure S8. sgRNA target sequence for editing *PtrHSFB3-1* and *PtrMYB092*.

Two sgRNAs were designed for CRISPR-based editing of *PtrMYB092* and *PtrHSFB3-1* in *P. trichocarpa.* The sgRNAs were designed to target the gene exons using the software CRISPR-P 2.0 (http://crispr.hzau.edu.cn/cgi-bin/CRISPR2/CRISPR) and IGV (http://software.broadinstitute.org/software/igv/). Number of base pairs (blue text) represents the locations of sgRNA target sites from the translational start site.

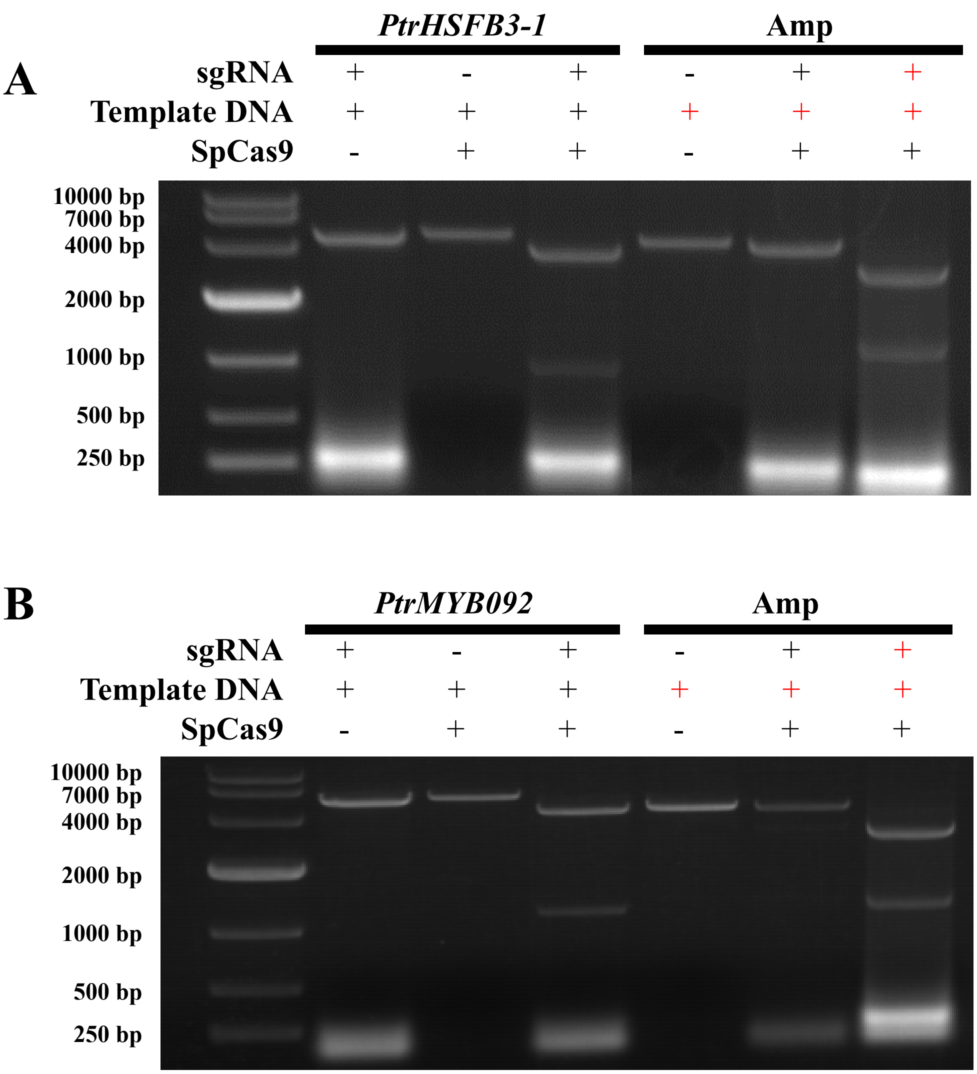

Supplemental Figure S9. Validation of sgRNAs using CRISPR RNP *in vitro* cleavage assays.

In vitro transcribed sgRNAs for *PtrHSFB3-1* (**A**) and *PtrMYB092* (**B**) were mixed with their respective target genes (template DNA) and recombinant SpCas9 to validate the cleavage efficiency of the sgRNAs *in vitro*. + represents the DNA template and sgRNA of the control gene ampicillin (Amp).

A

PtrHSFB3-1

Wildtype		GTGATATCATGGAACGGGGAGGG		
			Frame	
Mutant line 5-1*	Allele 1	GTGATATCATGGAACGG- -AGGG	-2 bp	Heterozygous
	Allele 2	GTGATATCATGGAACGGTGGAGGG	+1 bp	
Mutant line 5-2*	Allele 1	GTGATATCATGGAAATTACTTGGG	+1 bp	Heterozygous
	Allele 2	GTGATATCATGGAATGGA---GGG	-2 bp	
Mutant line 5-3*	Allele 1	GTGATATCATGGAATGGA---GGG	-2 bp	Homozygous
	Allele 2	GTGATATCATGGAATGGA---GGG		

B

PtrMYB092

Wildtype		AACCCTTGCACAAAGAAGCCAGG		
			Frame	
Mutant line 15-1*	Allele 1	AACCCTTGCACAAAGAAAGCCAGG	+1 bp	Homozygous
	Allele 2	AACCCTTGCACAAAGAAAGCCAGG		
Mutant line 15-2*	Allele 1	AACCCTTGCACAAAGAAGGCCAGG	+1 bp	Heterozygous
	Allele 2	AACCCTTGCACAAAGAAAGCCAGG		
Mutant line 15-3*	Allele 1	AACCCTTGCACAAAGAAGGCCAGG	+1 bp	Homozygous
	Allele 2	AACCCTTGCACAAAGAAGGCCAGG		

Supplemental Figure S10. CRISPR-based editing of *PtrHSFB3-1* and *PtrMYB092* in *P. trichocarpa.*

Mutations at the sgRNA target site for *PtrHSFB3-1* (**A**) and *PtrMYB092* (**B**) in the CRISPR-edited transgenic *P. trichocarpa*. The top rows (wildtype) denote the native sgRNA target sequence. Red letters represent the mutated nucleotides. Number of bp represent frame shifts after the mutations.

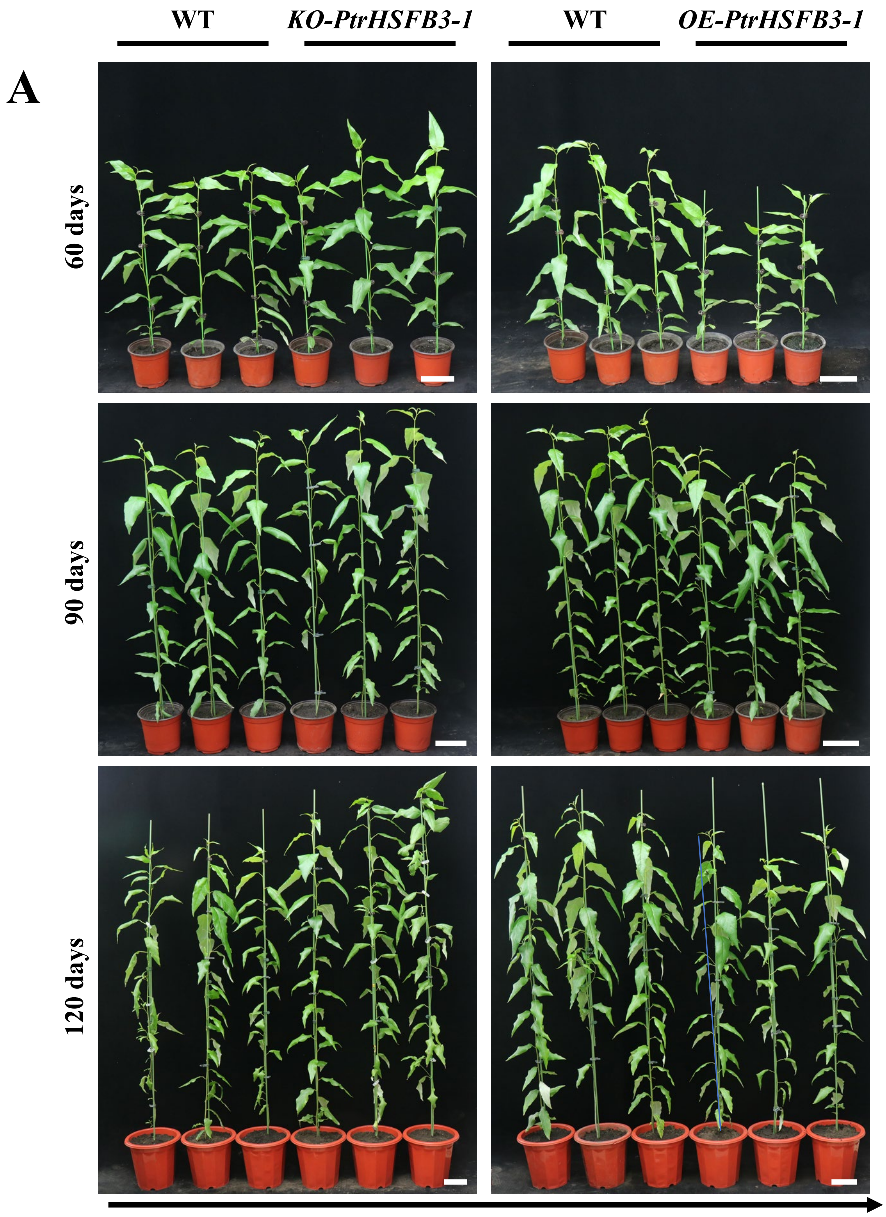

WT
KO-PtrHSFB3-1
WT
OE-PtrHSFB3-1
A
60 days
90 days
120 days

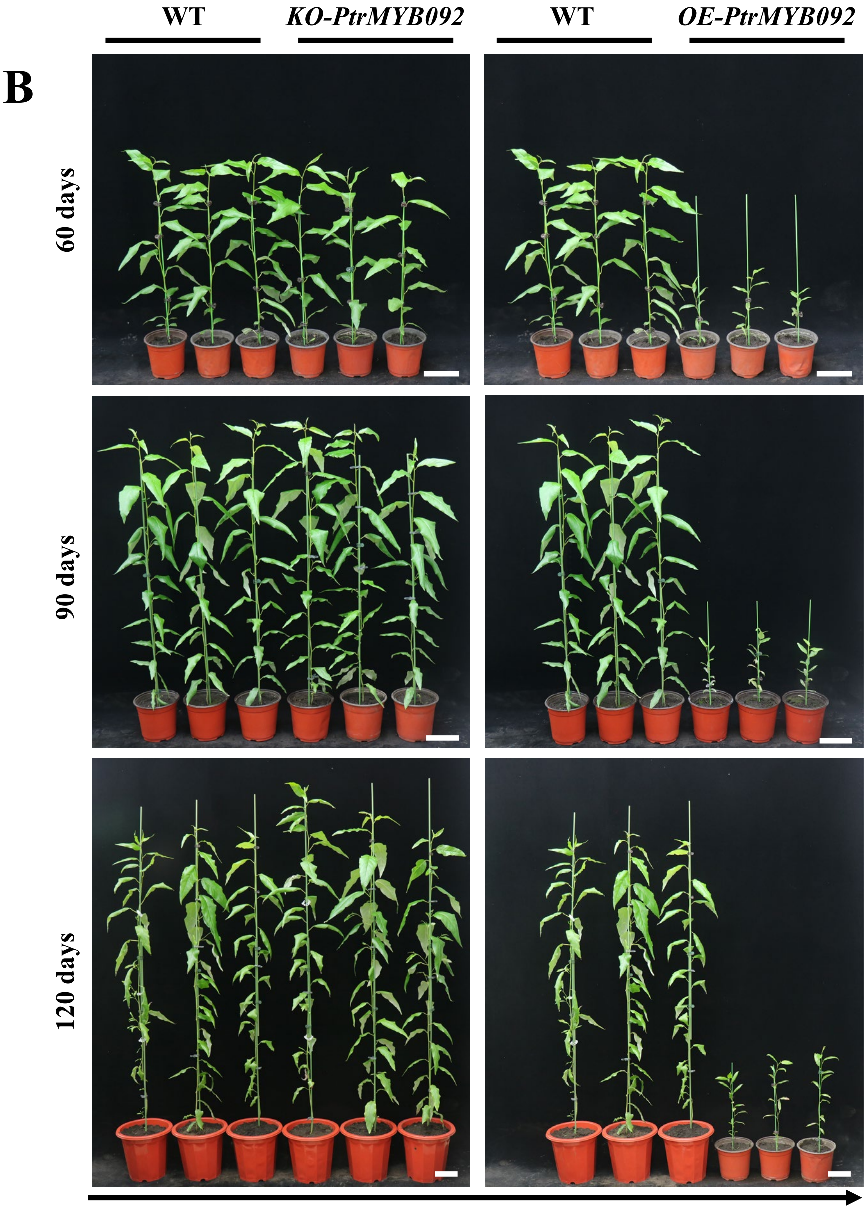

WT
KO-PtrMYB092
WT
OE-PtrMYB092
B
60 days
90 days
120 days

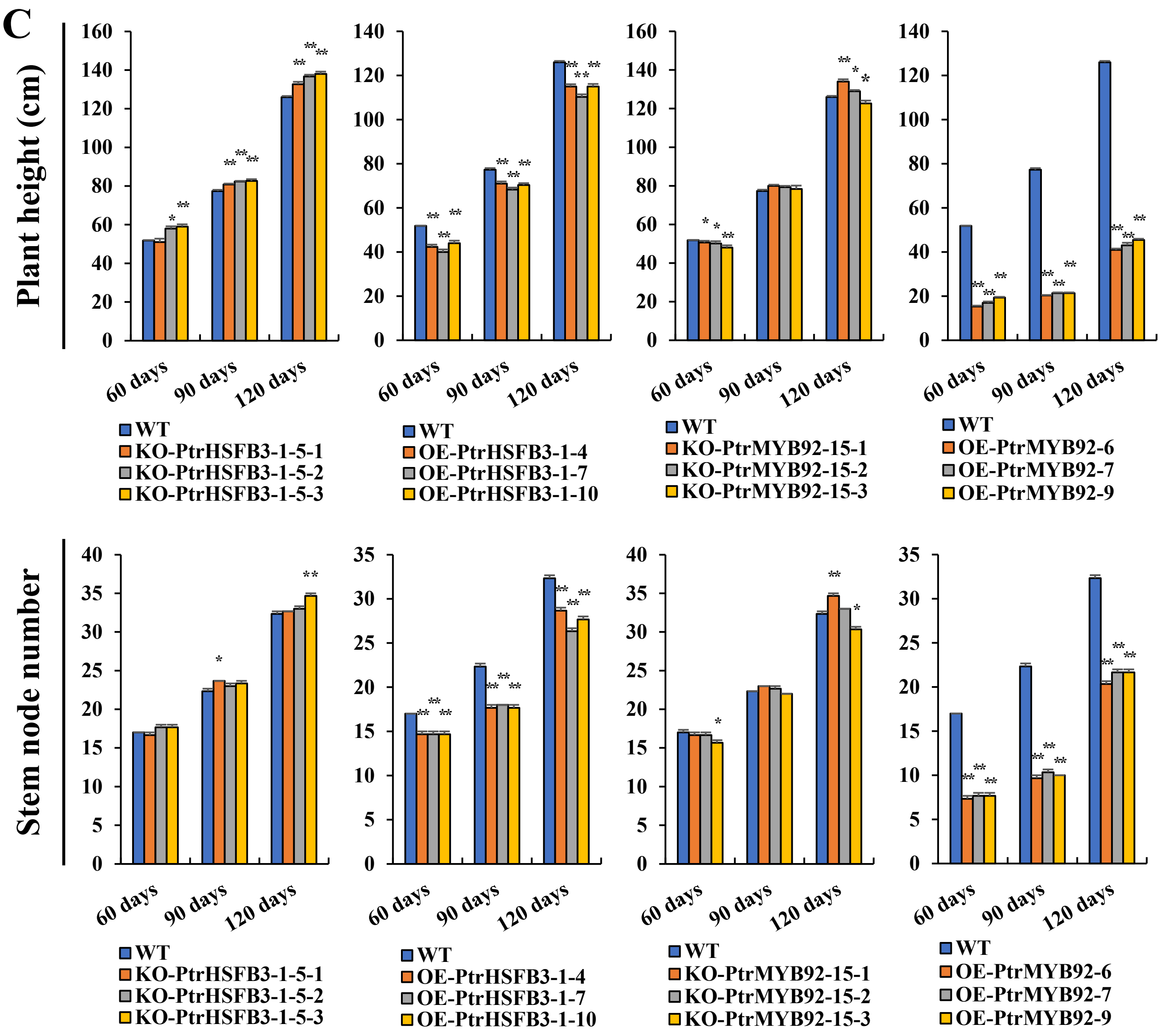

Supplemental Figure S11. Plant growth and number of stem internodes in transgenic *P. trichocarpa*.

Plants growing in an indoor greenhouse for 60, 90 and 120 days showed significant changes in height and number of internodes. Deleted expression of *PtrHSFB3-1* in *P. trichocarpa* significantly increased plant height after 90 and 120 days. Overexpression of *PtrHSFB3-1* and *PtrMYB092* in *P. trichocarpa* reduced plant height and number of stem internodes after 60, 90 and 120 days. (A) The phenotype of *KO-PtrHSFB3-1 and OE-PtrHSFB3-1*. The plants in the order indicated by the black arrow are WT1, 2, and 3, *KO-PtrHSFB3-1-5-1, -2* and *-3,* and *OE-PtrHSFB3-1-4, -7, and -11.* (B) The phenotype of *KO-PtrMYB092* and *OE-PtrMYB092*. The plants in the order indicated by the black arrow are WT1, 2, and 3, *KO-PtrMYB092-15-1, -2* and *-3,* and *OE-PtrMYB092-6*, *-7*, and *-9.* (C) Statistical analysis of plant height and number of stem internodes. Bars=10 cm. Error bars indicate one SE of three biological replicates (independent transgenic lines), and asterisks indicate significant difference between transgenics and WT for each gene, * $p < 0.05$ ** $p < 0.01$ (Student's t-test).

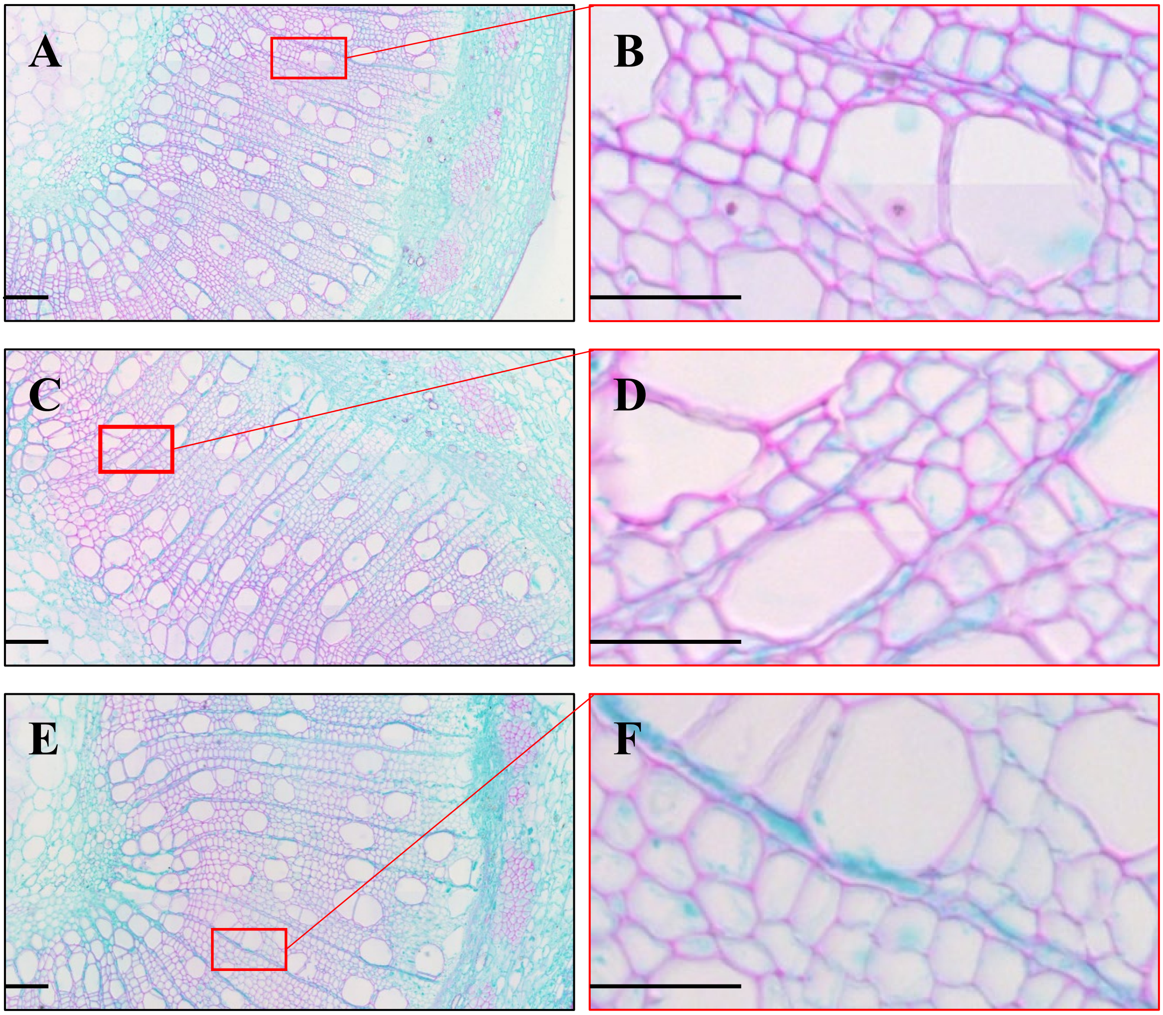

Supplemental Figure S12. Cell wall thickness in xylem of transgenic *P. trichocarpa*.

No significant changes were observed in cell walls of CRISPR-edited trees compared to wildtype (WT). Stem cross-sections of *KO-PtrHSFB3-1* (A-B) , *KO-PtrMYB092* (C-D) mutants and WT (E-F) at the 9th internode. Bars=100 µm.

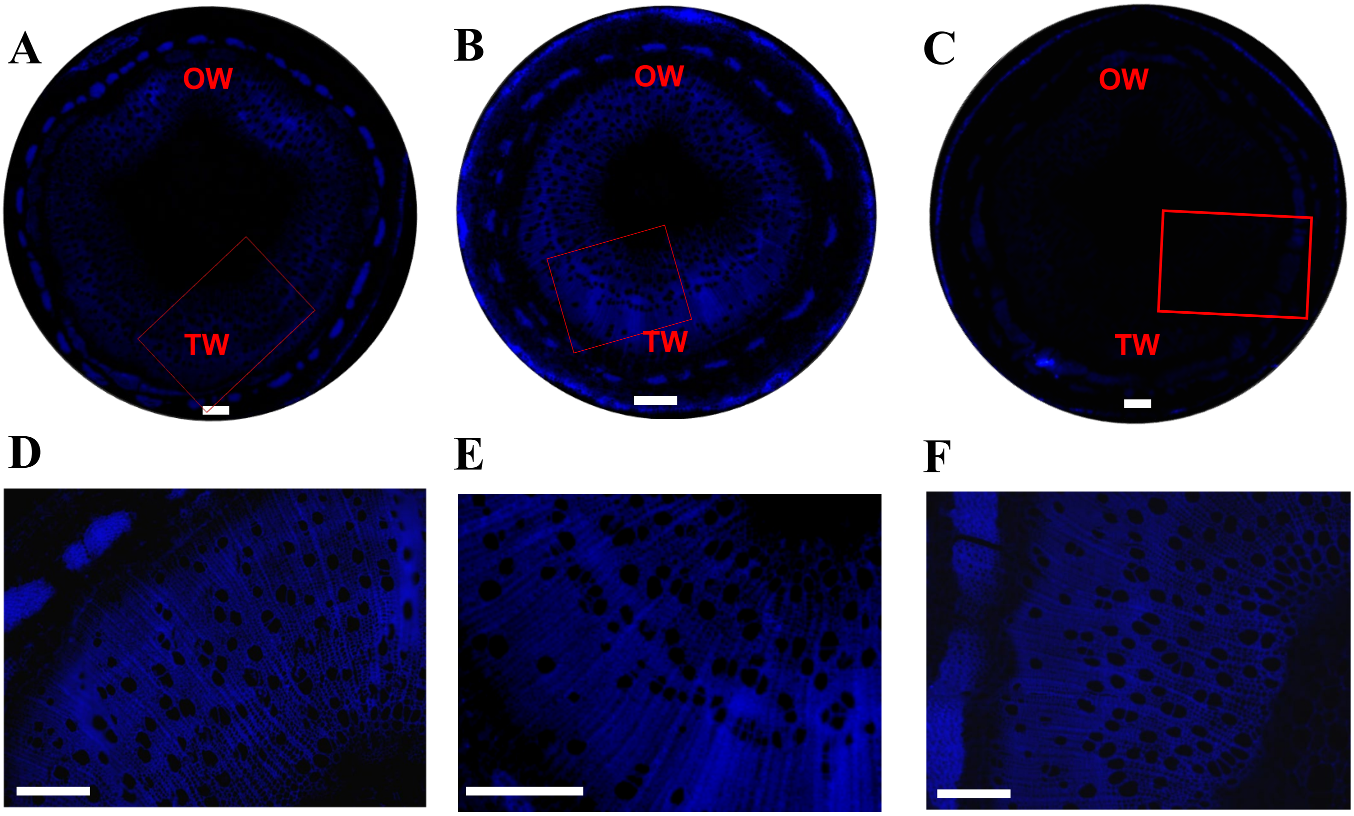

Supplemental Figure S13. Lignin autofluorescence imaging of OE-PtrMYB092, KO-PtrMYB092 transgenics and wildtype under tension stress induced by artificial stem bending. Images represent stem cross-sections (9th internode) of wildtype **(A, D),** OE-PtrMYB092 **(B, E),** and KO-PtrMYB092 **(C, F)**. Scale bars (A-F)= 200 µm. OW = opposite wood, TW = tension wood.

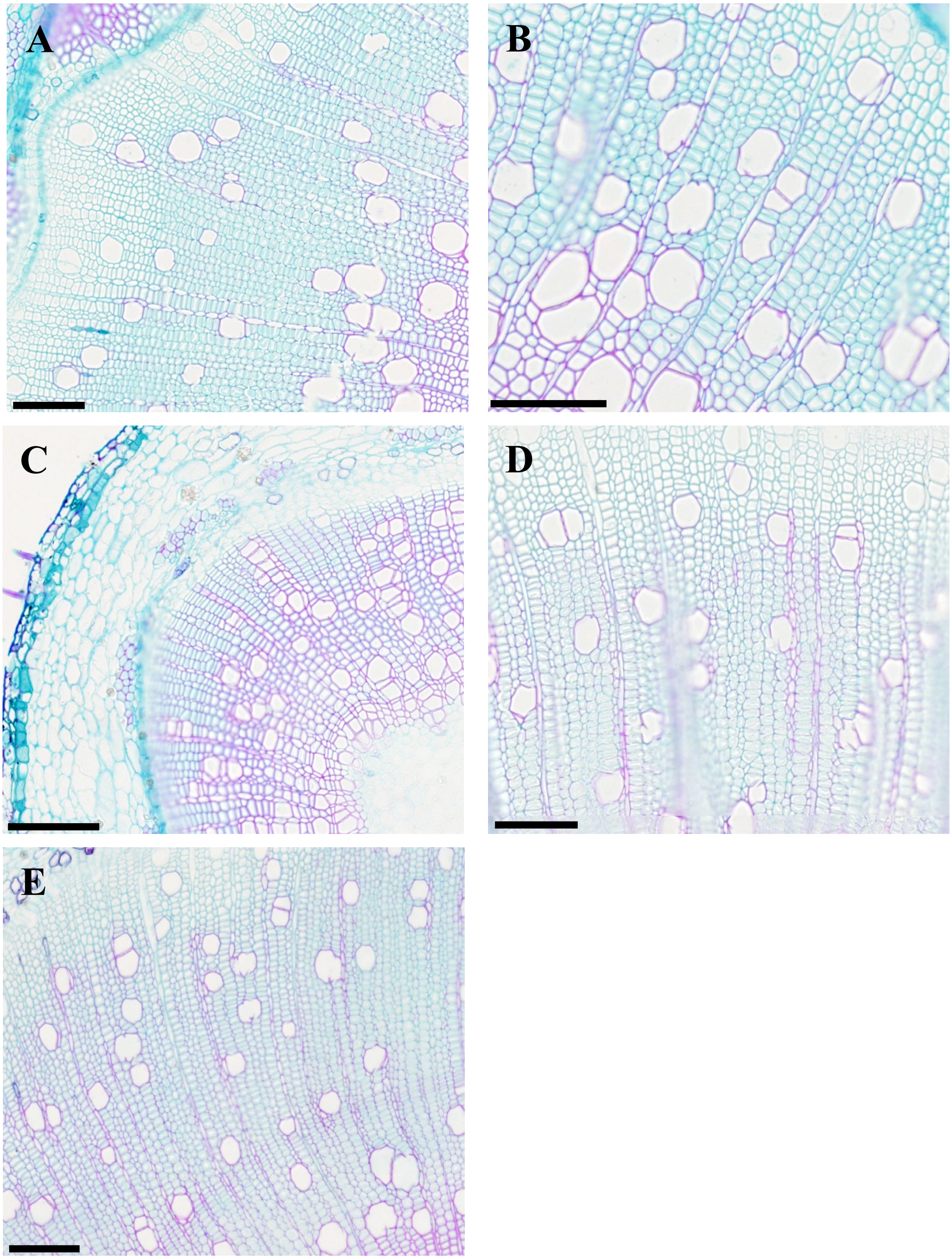

Supplemental Figure S14. Stem cross-section of tension-wood formation in wildtype, transgenic and mutant *P. trichocarpa*.

Stem cross-section of 3-month-old greenhouse grown *P. trichocarpa* 21 days after artificial bending to induce tension wood formation. Cross sections were stained with safranin O and fast green. Images represent stem cross-sections (9th internode) of wildtype **(A),** OE-PtrHSFB3-1 **(B),** OE-PtrMYB092 **(C)), KO**-PtrHSFB3-1 **(D)** and KO-PtrMYB092 **(E)**. Scale bars (O-S)= 100 µm.

Supplemental Table S1. Wood composition of vertical stem (WT) and tension wood of stem-bending for 21 days (**B-**WT) in wildtype *P. trichocarpa*

**Genotype**	**Wood sugar content** (g/100g of dry extractive-free wood)										**Wood lignin content** (g/100g of dry extractive-free wood)					
	**Glucose**		**Xylose**		**Galactose**		**Arabinose**		**Total carbohydrate**		**Acid insoluble lignin**		**Acid soluble lignin**		**Total lignin**	
	**Values**	**Average**	**Values**	**Average**	**Values**	**Average**	**Values**	**Average**	**Values**	**Average**	**Values**	**Average**	**Values**	**Average**	**Values**	**Average**
WT1	45.75		15.24		1.42		2.42		64.83		19.76		3.66		23.42	
WT2	46.29	45.72±0.34	15.35	15.33±0.05	1.60	1.54±0.06	2.30	2.17±0.19	65.55	64.77±0.47	20.38	20.08±0.18	3.48	3.54±0.06	23.86	23.62±0.13
WT3	45.12		15.41		1.60		1.80		63.93		20.10		3.48		23.58	
**B-**WT1	61.63		13.74		1.8		3.61		80.79		13.19		1.97		15.16	
**B-**WT2	57.85	59.71±1.09**	15.07	14.41±0.38**	2.26	2.01±0.38**	4.52	4.23±0.13**	79.71	80.26±0.32**	13.08	13.12±0.03**	2.08	2.03±0.21**	15.17	15.15±0.02**
**B-**WT3	59.65		14.40		1.97		4.25		80.28		13.08		2.02		15.10	

$p < 0.01$ (Student's t-test). ± Numbers indicate one standard error of three biological replicates. **B- indicate stem-bending for 21 days.

Supplemental Table S2. Percentage changes in wood composition of transgenics *P. trichocarpa* relative to wildtype

Genotype	**Wood sugar content** (g/100g of dry extractive-free wood)					**Wood lignin content** (g/100g of dry extractive-free wood)		
	**Glucose**	**Xylose**	**Galactose**	**Arabinose**	**Total carbohydrate**	**Acid insoluble lignin**	**Acid soluble lignin**	**Total lignin**
KO-PtrHSFB3-1	11.15±0.13	-5.62±2.39	-7.58±3.74	9.47±5.96	6.68±0.46	-16.81±0.89	-21.11±2.58	-17.46±0.52
KO-PtrMYB092	18.54±0.78	-5.65±1	-8.16±3.11	65.26±4.87	13.75±0.71	-27.91±0.55	-21.23±1.76	-26.91±0.69
OE-PtrHSFB3-1	-4.29±0.71	-4±0.31	-4±3.72	-4±8.35	-4.2±0.69	12.19±1.09	-4.2±4.61	9.73±0.24
OE-PtrMYB092	-7.49±1.11	0.05±0.15	-14.68±1.43	-27.85±3.68	-6.56±0.84	22.34±0.93	-5.48±1.6	18.17±0.58

± Numbers indicate one standard error of three biological replicates.

Supplemental Table S3. Percentage changes in tension wood composition of transgenics *P. trichocarpa* relative to wildtype

Genotype	**Wood sugar content** (g/100g of dry extractive-free wood)					**Wood lignin content** (g/100g of dry extractive-free wood)		
	**Glucose**	**Xylose**	**Galactose**	**Arabinose**	**Total carbohydrate**	**Acid insoluble lignin**	**Acid soluble lignin**	**Total lignin**
**B-***KO-PtrHSFB3-1*	9.05±1.05	-11.29±0.59	-14.37±4.10	-6.47±4.00	3.49±0.57	-18.76±2.11	3.51±1.93	-15.78±1.88
**B-***KO-PtrMYB092*	8.41±1.6	-8.76±3.46	37.86±8.17	28.45±2.96	7.09±0.61	-28.79±3.29	-14.59±4.94	-26.89±2.26
**B-***OE-PtrHSFB3-1*	-3.38±1.24	-8.52±1.73	-28.70±2.04	-30.45±1.99	-6.33±0.50	14.25±1.15	6.5±1.40	13.2±1.08
**B-***OE-PtrMYB092*	-20.94±1.51	-12.61±3.78	-31.31±4.57	-33.00±4.46	-20.32±64	37.19±1.02	3.37±1.95	32.67±1.13

± Numbers indicate one standard error of three biological replicates. **B-** indicate stem-bending for 21 days.
